# The genetic regulation of protein expression in cerebrospinal fluid

**DOI:** 10.15252/emmm.202216359

**Published:** 2022-12-12

**Authors:** Oskar Hansson, Atul Kumar, Shorena Janelidze, Erik Stomrud, Philip S Insel, Kaj Blennow, Henrik Zetterberg, Eric Fauman, Åsa K Hedman, Michael W Nagle, Christopher D Whelan, Denis Baird, Anders Mälarstig, Niklas Mattsson‐Carlgren

**Affiliations:** ^1^ Clinical Memory Research Unit, Faculty of Medicine Lund University Lund Sweden; ^2^ Memory Clinic Skåne University Hospital, Lund University Lund Sweden; ^3^ Department of Psychiatry and Behavioral Sciences University of California, San Francisco San Francisco CA USA; ^4^ Clinical Neurochemistry Laboratory Sahlgrenska University Hospital Mölndal Sweden; ^5^ Department of Psychiatry and Neurochemistry, Institute of Neuroscience and Physiology, the Sahlgrenska Academy University of Gothenburg Mölndal Sweden; ^6^ Department of Neurodegenerative Disease UCL Institute of Neurology London UK; ^7^ UK Dementia Research Institute at UCL London UK; ^8^ Hong Kong Center for Neurodegenerative Diseases Hong Kong China; ^9^ Internal Medicine Research Unit Pfizer Worldwide Research, Development and Medical Cambridge MA USA; ^10^ Pfizer Worldwide Research, Development and Medical Stockholm Sweden; ^11^ Department of Medical Epidemiology and Biostatistics Karolinska Institutet Stockholm Sweden; ^12^ Neurogenomics, Genetics‐Guided Dementia Discovery Eisai, Inc Cambridge MA USA; ^13^ Translational Biology, Biogen Research & Development Biogen Inc Cambridge MA USA; ^14^ Department of Neurology, Skåne University Hospital Lund University Lund Sweden; ^15^ Wallenberg Center for Molecular Medicine Lund University Lund Sweden

**Keywords:** biomarkers, cerebrospinal fluid, genetic regulation, Mendelian randomization, pQTL, Genetics, Gene Therapy & Genetic Disease, Neuroscience, Proteomics

## Abstract

Studies of the genetic regulation of cerebrospinal fluid (CSF) proteins may reveal pathways for treatment of neurological diseases. 398 proteins in CSF were measured in 1,591 participants from the BioFINDER study. Protein quantitative trait loci (pQTL) were identified as associations between genetic variants and proteins, with 176 pQTLs for 145 CSF proteins (*P* < 1.25 × 10^−10^, 117 *cis*‐pQTLs and 59 *trans*‐pQTLs). Ventricular volume (measured with brain magnetic resonance imaging) was a confounder for several pQTLs. pQTLs for CSF and plasma proteins were overall correlated, but CSF‐specific pQTLs were also observed. Mendelian randomization analyses suggested causal roles for several proteins, for example, ApoE, CD33, and GRN in Alzheimer's disease, MMP‐10 in preclinical Alzheimer's disease, SIGLEC9 in amyotrophic lateral sclerosis, and CD38, GPNMB, and ADAM15 in Parkinson's disease. CSF levels of GRN, MMP‐10, and GPNMB were altered in Alzheimer's disease, preclinical Alzheimer's disease, and Parkinson's disease, respectively. These findings point to pathways to be explored for novel therapies. The novel finding that ventricular volume confounded pQTLs has implications for design of future studies of the genetic regulation of the CSF proteome.

## Graphical abstract

The genetic regulation of cerebrospinal fluid (CSF) proteins can be explored to increase the understanding of brain disease mechanisms. This study explored protein quantitative trait loci (pQTLs) for 398 CSF proteins analyzed by highly specific protein extension assays in a large human population.

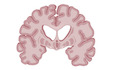

## Introduction

Cerebrospinal fluid (CSF) is produced within the brain and is a rich source for biomarkers that correlate with brain pathologies across different diseases (Hansson, 2021). While studies of the genetic control of the CSF proteome may yield insights into brain disease mechanisms and potential treatments, only a few such studies have been performed to date on larger sets of proteins (Kunkle *et al*, 2019; Sasayama *et al*, 2019; Yang *et al*, 2021), or focused on a few specific disease‐associated proteins (Deming *et al*, 2017; Maxwell *et al*, 2018). In contrast, several studies in blood plasma have consistently shown that genetic variants associated with protein levels, known as protein quantitative trait loci (pQTL), using both aptamer‐based (Sun *et al*, 2018; Ferkingstad *et al*, 2021) and antibody‐based assays (Folkersen *et al*, 2020) are common and can explain up to 30% of the protein variance. Identification of pQTLs makes it possible to test if proteins are likely to be causal for development of human diseases (hence nominating them as candidate drug targets). For neurological diseases, more pQTL studies are needed on proteins in CSF rather than plasma to identify disease pathways and potential drug targets. The largest previous study on CSF pQTLs used an aptamer‐based approach (Yang *et al*, 2021). Recent comparisons between aptamer‐ and antibody‐based pQTL studies highlight that the technology used for protein quantification could have a significant impact on the findings, with antibody‐based methods being more specific (Katz *et al*, 2022). Therefore, we attempted to discover both CSF and plasma pQTLs in a large and deeply phenotyped cohort of healthy controls and patients with different neurological diseases, using highly specific biomarker assays to capture a wide range of biological pathways. We mainly used proximity extension assays (PEA), which have been shown to be sensitive and specific (Assarsson *et al*, 2014; Katz *et al*, 2022), together with selected other assays, including for orthogonal validation. We also evaluated ventricular volume measured with magnetic resonance imaging (MRI) as a potential confounder (due to dilution effects) for CSF pQTLs, which to our knowledge had not been done before in CSF pQTL studies. The overarching aim of the study was to map and decipher clinically relevant genetic regulation of proteins expressed in the brain and subsequently secreted to the CSF, with a focus on brain diseases in general and neurological diseases in particular.

## Results

### Genome‐wide analysis of 398 CSF proteins reveals 176 independent genetic loci associated with CSF levels of 145 proteins

**Figure 1:**
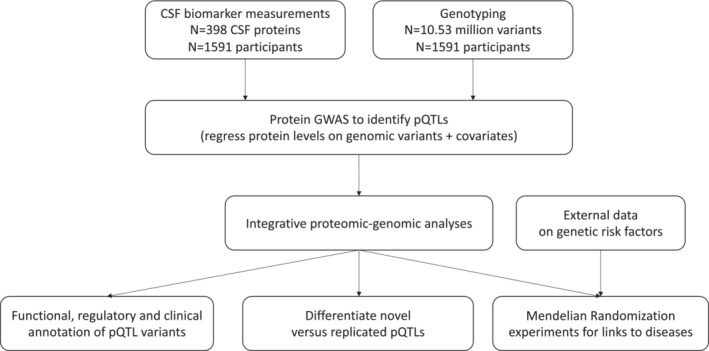
Study flowchart A schematic overview of the study design.

The overall study design is shown in Fig 1. In total, 1,591 study participants were included (53% females), with a mean age of 71.3 (standard deviation 7.1) years. Most participants were cognitively unimpaired (CU) individuals (613 cognitively normal controls and 210 individuals with subjective cognitive decline [SCD]), and the remaining were patients with different diseases, including 280 mild cognitive impairment (MCI) patients, 189 Alzheimer's disease (AD) dementia patients, 155 Parkinson's disease (PD) patients, 30 Parkinsons' disease dementia (PDD) patients, 21 progressive supranuclear palsy (PSP) patients, 24 dementia with Lewy bodies (DLB) patients, 14 vascular dementia (VaD) patients, 5 frontotemporal lobe dementia (FTD) patients, 27 multiple system atrophy (MSA) patients, 6 corticobasal syndrome (CBS) patients, 11 patients with unspecified parkinsonism, and 6 patients with unspecified dementia (demographics by diagnosis is shown in Table EV1).

**Figure 2:**
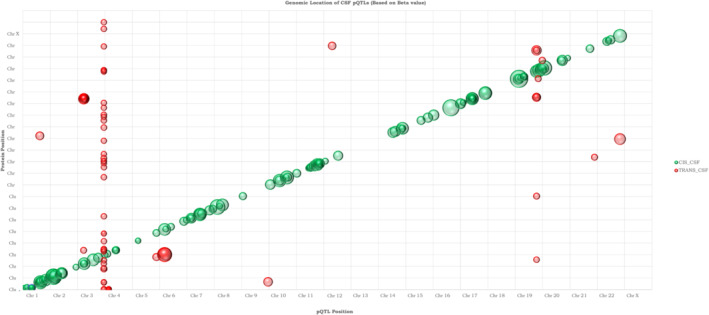
CSF pQTL genomic map Each point represents a significant pQTL for a CSF protein. The sizes of the bubbles are proportional to the β‐coefficient of the effects. An interactive version of the plot is available as source data for Fig EV1.

**Figure 3:**
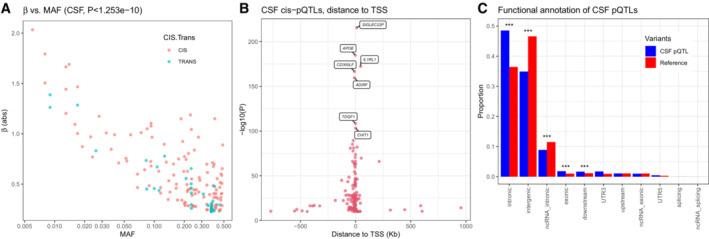
CSF pQTL mapping Relationships between pQTL effect (β) and minor allele frequency (MAF) for CSF pQTLs. *cis*‐pQTLs and *trans*‐pQTLs are indicated (only pQTLs significant after Bonferroni correction are included).For all Bonferroni significant CSF *cis*‐pQTLs, the degree of significance is shown by the distance from transcription start site (TSS). The interquartile range was −37.3 to 0.12 Kb. The most significant pQTLs are annotated.Functional annotation of genetic variants for CSF pQTLs, generated by FUMA, for CSF pQTLs that were significant after Bonferroni correction. Enrichment of functional consequences of SNPs was tested against the European 1,000 genome reference panel. Asterixes indicate significant differences between proportions for significant CSF pQTLs versus the reference panel (***P < 0.001; *P < 0.05).

**Figure EV1:**
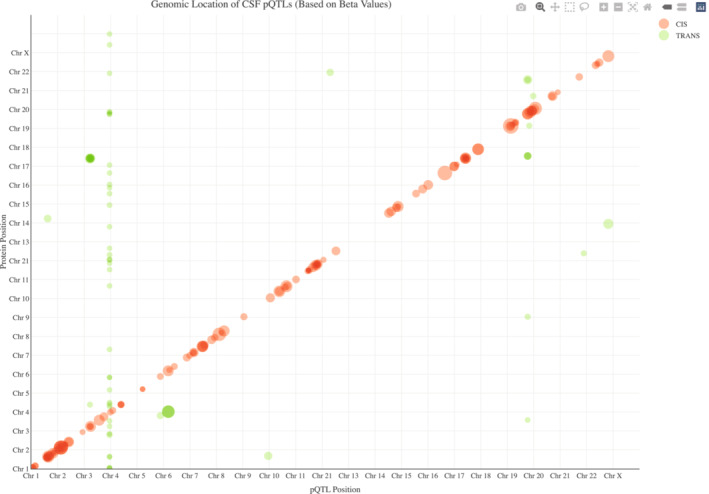
Interactive pQTL genomic map for CSF pQTLs This is an interactive version of main Fig 2. Full interactive functionality is provided in the Source Data file “Fig EV1 CSF_pQTL_interactive.html”, which is available online. Source data are available online for this figure.

There were 398 proteins in CSF available (Dataset EV1) for genome‐wide analyses using 10.53 million common genetic variants (with imputation INFO score ≥ 0.6; MAF ≥ 1%). After correction for multiple comparisons using the Bonferroni method (genome‐wide significance *P* < 5 × 10^−8^ divided by number of tested proteins, *P* < 1.253 × 10^−10^ for CSF), we found 176 significant CSF pQTLs among 145 proteins (Figs 2 and EV1), including cis‐pQTLs (*N* = 117) and *trans*‐pQTLs (*N* = 59). We also found 197 plasma pQTLs (*N* = 141 *cis*‐pQTLs and *N* = 56 *trans*‐pQTLs), among 153 proteins. These main results are in Dataset EV2 (together with results for all pQTLs with at least genome‐wide significant associations [*P* < 5 × 10^−8^] included to facilitate future meta‐analyses). The pQTLs were distributed across the different assay platforms used (Appendix Fig S1). The minor allele frequencies of the pQTLs were inversely correlated with effect size (Fig 3A), and the strengths of the associations for *cis*‐pQTL decreased at longer distance from transcription start sites (Fig 3B). We performed functional annotation of the CSF variants (Fig 3C) and found enrichment (after Bonferroni correction over 11 categories) for intronic (49% for CSF variants vs. 36% in a reference panel), exonic (1.8% vs. 1.0%), and downstream (1.7% vs. 1.1%) variants, while intergenic (35% vs. 47%) and ncRNA‐intronic (8.9% vs. 11.5%) were significantly less common.

**Figure EV2:**
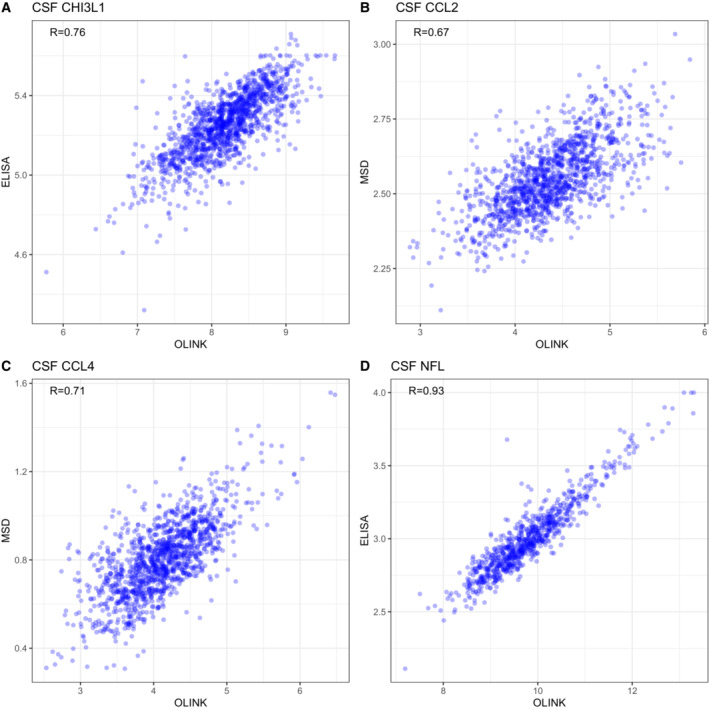
CSF biomarkers measured with orthogonal methods A–D Between‐assay correlations for CSF biomarkers measured with both OLINK methods (proximity extension assay) and orthogonal methods, for four proteins where this data was available (panel A: CHI3L1, panel B: CCL2, panel C: CCL4, panel D: NFL). pQTLs were identified for CHI3L1, CCL2 and CCL4, as described in the main manuscript. pQTLs identified by the orthogonal methods are included in Dataset EV2 (using alternative protein labels for the alternative assays: MCP1 for CCL2, MIP1b for CCL4, and YKL‐40 for CHI3L1, rows marked yellow). For CCL2, the same genetic variant was identified (rs2228467, *trans*‐pQTL) with both assays. For CCL4, one *trans*‐pQTLs identified by proximity extension assay was validated (rs113341849) and one *cis*‐pQTL (rs879571071) was identified, which was in LD with a *cis*‐pQTL identified for the proximity extension assay (rs8064426, R^2^ = 0.200, D′ = 0.687). For CHI3L1, one *cis*‐pQTL was also identified (rs4950928), which was in high LD with a *cis*‐pQTL identified for the proximity extension assay (rs946262, R^2^ = 0.902, D′ = 1.0). For all these cases, the effect sizes of the pQTLs were similar, with stable direction of effects.

The genome‐wide analyses were adjusted for diagnostic group, but in a sensitivity analysis, we repeated the CSF analyses in the subgroup of cognitively normal controls, with similar results as the main analysis (Appendix Fig S2). For three proteins with significant CSF pQTL findings, we had measures with both PEA and additional assays (Meso Scale Discovery for CCL2 and CCL4, and ELISA for CHI3L1), and the pQTL findings were validated using the alternative assays (Fig EV2; Dataset EV2, lines marked in yellow). Our main method to annotate pQTL genes was by genomic distance, but annotation through chromatin interaction analysis yielded largely overlapping results (Dataset EV2).

**Figure EV3:**
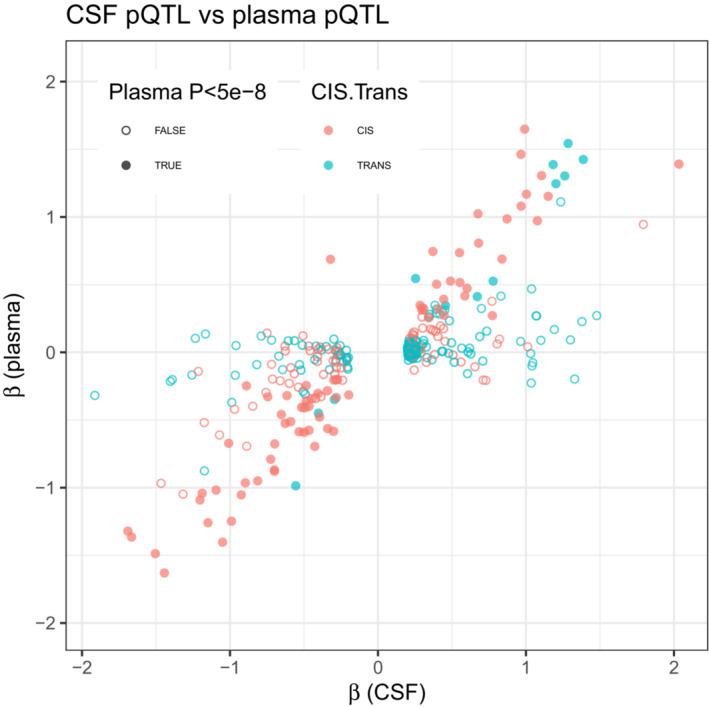
pQTLs in CSF and plasma The figure shows the general relationship between pQTLs in CSF and plasma, for all pQTLs that were at least genome‐wide significant (*P* < 5e‐8) in CSF. The only pQTL which was significant after Bonferroni correction in CSF and genome‐wide significant in plasma, where the direction of effect differed between the tissues, was a cis‐pQTL for CXCL1, where the variant was associated with lower levels in CSF and higher in plasma.

In general, CSF and plasma pQTL were highly correlated (Spearman Rho = 0.69, *P* < 0.001, Fig EV3). However, out of 162 Bonferroni significant CSF pQTLs where the matching protein was also analyzed in plasma, there were 74 (46%) pQTLs (44 *cis*‐pQTLs and 43 *trans*‐pQTLs, distributed among 39 putative genes) which were non‐significant even at genome‐wide level (*P* > 5 × 10^−8^) in plasma. One example was IL‐6, with a *cis*‐pQTL (rs79103996) that was highly significant in CSF (*P* = 2.9 × 10^−29^) but had no association in plasma (*P* = 0.38), suggesting relevant tissue‐specificity of IL‐6 regulation. When testing for tissue enrichment among the genes with putative CSF‐specific pQTLs, two genes were enriched in cerebral cortex (*OSTN*, *N* = 34 *trans*‐pQTLs and *DRAXIN*, *N* = 1 *cis*‐pQTL).

### Genetic variant‐to‐protein specificity

**Figure 4:**
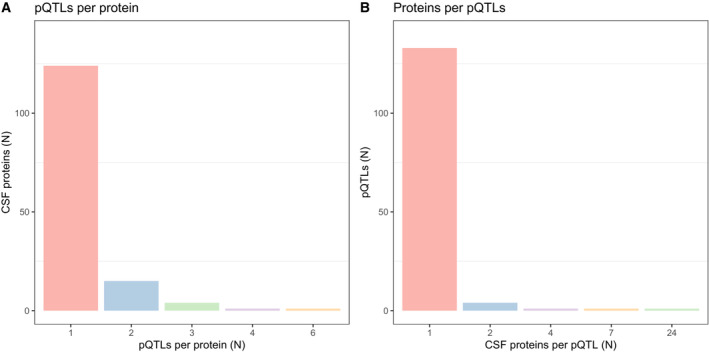
Overview of CSF pQTL architecture Number of pQTLs per CSF protein (panel A), and number of CSF proteins per top genetic variant among the identified pQTLs (panel B).

Among all significant (after Bonferroni correction) CSF pQTLs, there were 140 unique genetic loci and 145 unique proteins. Most CSF proteins had only one locus, predominantly acting in *cis* (Fig 4A, see Appendix Fig S3 for plasma pQTLs), but a few proteins had several *trans*‐acting loci (for all identified loci, the strongest pQTL was nominated and carried on further in all analyses), including six loci for CSF CCL4 (two *cis*‐pQTLs and four *trans*‐pQTLs), four for CSF RBKS (all *cis*‐pQTLs), and three each for CSF CCL24 (all *cis*‐pQTLs), CTSS (all *cis*‐pQTLs), and LRPAP1 (all *trans*‐pQTLs). Conversely, most genetic variants among the significant pQTLs were only associated with one CSF protein (Fig 4B). One notable exception was the genomic region represented by variant rs71635338 which was significantly associated with 24 different CSF proteins (all *trans*‐pQTLs). This variant was in an intergenic region on chromosome 3, where other *trans*‐pQTLs were also nominated for some proteins (see below for a detailed analysis). We noted that the variant rs429358 was associated with multiple CSF proteins (*N* = 6 *trans*‐pQTLs and *N* = 1 *cis*‐pQTLs). This genetic variant defines the *APOE* genotype, which has well‐known associations with CSF biomarkers related to Alzheimer's disease (AD), which were validated here (lower Aβ42, higher T‐tau and P‐tau (Chung *et al*, 2018)).

### Validation of previously identified pQTLs

We queried the EBI‐GWAS catalog (September 2022) for all Bonferroni significant CSF pQTLs identified in this study, as described in the Methods section. Novelty of the CSF pQTLs was assessed on multiple levels, depending on whether we found in the GWAS catalog any prior pQTL in the locus, a prior pQTL for the protein we observed at the locus, any prior pQTL in CSF at the locus, or a prior pQTL in CSF for the protein we observed at the locus. The result of this novelty assessment is provided in Dataset EV3. A full summary of the GWAS catalog hits is provided in Dataset EV4. Detailed results for CNS disease hits and pQTL hits are provided in Datasets EV5 and EV6, respectively. In summary, among all CSF pQTL‐protein pairs included in this analysis, only 19 (10.9%) were validated from previous publications (the pQTL was reported before for the same protein in CSF), 82 (47.1%) were novel in CSF but a pQTL in the same region had been reported before for the same protein in serum or plasma, 43 (24.7%) were novel for the identified protein, but the locus had been associated before with other proteins in CSF, 27 (15.5%) were novel for the identified protein, but the locus had been associated before with other proteins in serum or plasma, and 3 (1.7%) were completely novel (no pQTL reported before for the locus for any protein in any tissue).

**Figure EV4:**
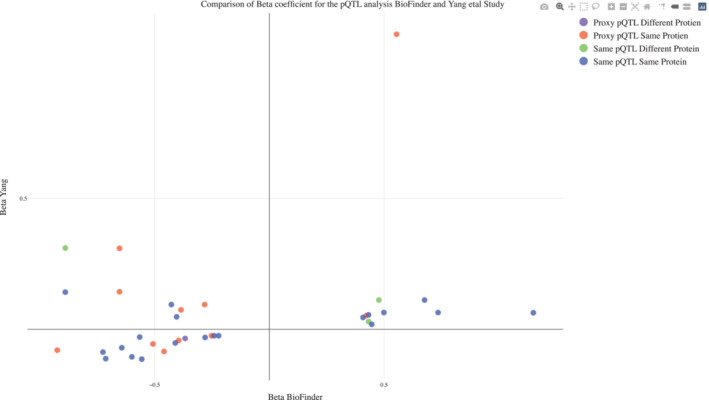
Replication of CSF pQTLs from Yang *et al* (2021) This figure shows a comparison between pQTLs that were identified both in the current study and in another recent publication on CSF pQTLs (Yang *et al*, 2021). Full interactive functionality is provided in the Source Data file “Fig EV4 Replication CSF pQTLs.html”, which is available online. Source data are available online for this figure.

Another large study has recently been published on CSF proteins and genetic variants (Yang *et al*, 2021), which used an aptamer‐based proteomics technology on 971 participants with CSF samples, and identified 275 CSF pQTLs. Our studies applied different CSF protein panels but overlapped for 235 proteins. We conducted a head‐to‐head comparison for overlapping CSF pQTL results. Fifteen pQTL‐protein pairs were replicated (significant effects with similar direction of effect with respect to the risk allele) between the studies (see Fig EV4; Dataset EV7). Out of these 15, 12 pQTL‐protein pairs were *cis*‐acting and three were *trans*‐acting. Further, we identified 8 cis pQTL‐protein pairs with a proxy match (up to 10 kb) with Yang *et al* (2021) pQTL‐protein pairs (with the same protein and same directionality in the two studies). We also identified two additional pQTLs that were common in both studies but associated with different proteins. One of these (rs76904798) was *trans*‐acting and was associated with GPNMB in our study and GRN in Yang *et al* (2021). The second pQTL (rs12126142) was *trans*‐acting in our study (PSME1) but *cis*‐acting in the Yang *et al* (2021) study (IL6R). We also noted that there were eight *cis*‐pQTLs (including three with the same genetic variant and same protein, four with proxy match pQTL and same protein, and one with the same genetic variant but different proteins) overlapping between the two studies, but where the direction of effect was reversed between the studies (lower part of Dataset EV7).

### Matching and co‐localization of cis‐pQTLs and eQTLs

**Figure 5:**
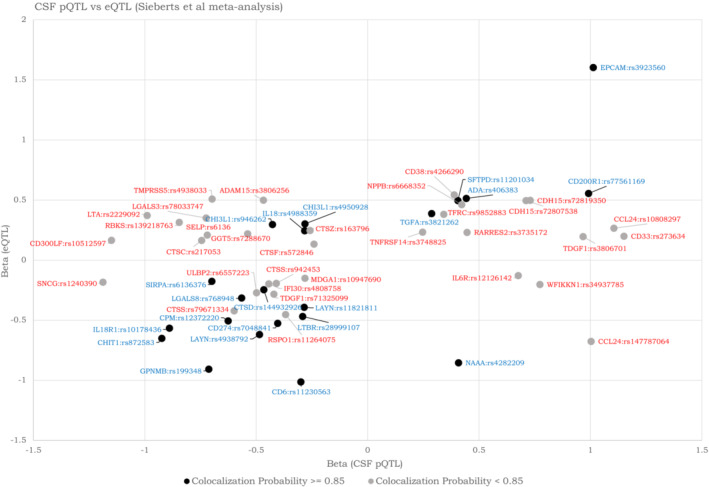
eQTLs and CSF pQTLs for corresponding genetic variants and proteins The plot includes significant CSF pQTLs and eQTLs, from the meta‐analysis by Sieberts *et al*, 2020. Most of the significant variants had concordant directions as eQTLs and pQTLs. Co‐localization is indicated by color of text and shapes (probability 0.85 or higher shown in blue with dark shapes, probability below 0.85 shown in red with gray shapes).

To further understand the mechanism by which genetic variants influence CSF protein levels, we investigated the effects of pQTLs on mRNA gene expression. All Bonferroni significant CSF *cis*‐pQTLs were queried both in a large eQTL meta‐analysis study (Sieberts *et al*, 2020) and in the GTEx eQTL database (restricting the search to brain tissues), using a *P*‐value of *P* < 0.05 (false discovery rate [FDR]‐adjusted) to denote statistical significance. When comparing with the eQTL meta‐analysis study, 53 of the *cis*‐pQTLs identified in the current study showed previous evidence of association with gene expression in brain tissues. The effect direction was concordant for 35 and discordant for 18 of the pQTLs (Fig 5; Dataset EV8). Co‐localization experiments showed that concordant QTLs tended to have higher co‐localization probability (18/35 had co‐localization probability of at least 0.85), while discordant QTLs tended to have lower co‐localization probability (only 4/18 had co‐localization probability of at least 0.85). The GTEx eQTL database query resulted in identification of 7 additional *cis*‐pQTLs (not found in the meta‐analysis study), where the effect direction was concordant for 5 and discordant for 2 pQTLs (Appendix Fig S4 and Dataset EV9).

### Role of trans‐pQTLs

We also attempted to nominate a causal gene for the CSF *trans*‐pQTLs, searching for evidence of links between genes located close to the *trans*‐pQTL and the protein‐encoding gene. For the Bonferroni significant CSF *trans*‐pQTLs, we searched the STRING/KEGG database for such links. Each gene–gene pair was given an interaction score based on co‐expression, experimentally determined interactions, and database annotation. Seven of the CSF *trans*‐pQTLs reached the *a priori* determined interaction score limit (> 0.7), indicating previous evidence of significant gene‐to‐gene links. These included CSF Aβ42, P‐tau, and T‐tau (for *APOE*), CCL4 (for *CCR2* and *CCR9*), IL12A (for *IL12B*), and TNFSF13B (for *TNFRSF13C*; Appendix Fig S5).

### Shared genetic effects between pQTLs and neuropsychiatric traits

To test if there was evidence for sharing genetic effects between pQTLs and brain‐related disorders we conducted a combination of Mendelian randomization (MR) and genetic colocalization analysis. For the MR, we performed Wald ratio (WR) tests based on the independent *cis*‐pQTL instruments available for each protein. The WR determines the change in disease risk per SD change in protein levels explained through the risk allele of the instrumenting pQTL. Therefore, a positive WR would indicate that genetically predicted increased protein expression is related to disease risk, whereas a negative WR would indicate that decreased protein expression is related to disease risk. To conduct this MR analysis, we combined our pQTL results with external GWAS summary data for 32 different outcomes, including neurological diseases and brain‐related traits (Dataset EV10).

The MR‐results for *cis*‐pQTLs are summarized in Table 1 and Dataset EV11. After Bonferroni correction (over the entire set of MR analyses), there were 18 pQTL‐protein pairs with strong WR evidence for sharing a genetic effect across 13 different outcomes. The APOE pQTL (rs429358) had shared effects with AD, preclinical AD, amygdala volume, hippocampus volume, and nucleus accumbens volume. The CD33 pQTL (rs273634) and GRN pQTL (rs5848) had shared effects with AD. The MMP10 pQTL (rs11225415) had shared effects with preclinical AD. CTSF was associated with one pQTL (rs572846) and had shared effects with bipolar disorder and depression. The SIGLEC9 pQTL (rs2258983) had shared effects with amyotrophic lateral sclerosis. We also observed two protein‐pQTL pairs (FLRT2‐rs17646457 and RARRES2‐rs3735172) with shared effects with focal epilepsy, as well as hippocampal sclerosis‐related epilepsy. Three different pQTL‐protein pairs had shared effects with PD, including CD38‐rs4266290, GPNMB‐rs199348, and ADAM15‐rs3806256. The pQTL‐protein pairs CTSS‐rs79671334 and RARRES2‐rs3735172 had shared effects with schizophrenia and year of schooling, respectively. We queried the Cortellis Integrity database for *cis*‐pQTL proteins identified in the MR analyses and found that drug candidates were available for APOE, CD33, GRN, CD38, ADAM15, and CTSS (Table 2).

**Table 1:** Mendelian randomization experiments for *cis*‐pQTLs.

SNP	Exposure	Outcome	Wald ratio	*SE*	*P*	*P* (Bonf.)	Type	Co‐localization probability
rs429358	APOE	AD	−1.13	0.04	0	0	*CIS*	1
rs273634	CD33	AD	0.04	0.01	2.75E‐06	2.64E‐04	*CIS*	0.78
rs5848	GRN	AD	−0.21	0.06	2.33E‐04	2.24E‐02	*CIS*	0.99
rs429358	APOE	AD (Preclinical)	−0.1	0.006	7.5E‐120	7.8E‐118	*CIS*	0.81
rs11225415	MMP10	AD (Preclinical)	0.05	0.01	3.5E‐04	3.6E‐02	*CIS*	6.6E‐07
rs429358	APOE	Amygdala volume	14.04	3.27	1.77E‐05	1.63E‐03	*CIS*	0.99
rs2258983	SIGLEC9	Amyotrophic Lateral Sclerosis	0.12	0.03	1.18E‐04	1.10E‐02	*CIS*	0.93
rs572846	CTSF	Bipolar Disorder	−0.25	0.07	1.10E‐04	1.05E‐02	*CIS*	0.11
rs572846	CTSF	Depression	−0.02	0.01	3.82E‐04	3.62E‐02	*CIS*	0.035
rs17646457	FLRT2	Focal Epilepsy	−0.07	0.02	5.27E‐04	3.32E‐02	*CIS*	0.03
rs3735172	RARRES2	Focal Epilepsy Hippocampal Sclerosis	−0.01	0.004	6.83E‐04	4.51E‐02	*CIS*	0.35
rs429358	APOE	Hippocampus volume	23.59	6.57	3.33E‐04	3.06E‐02	*CIS*	0.87
rs429358	APOE	Nucleus accumbens volume	5.77	1.48	1.00E‐04	9.24E‐03	*CIS*	0.95
rs4266290	CD38	PD	−0.33	0.06	6.28E‐08	5.97E‐06	*CIS*	0.52
rs199348	GPNMB	PD	0.14	0.03	9.57E‐08	9.09E‐06	*CIS*	0.94
rs3806256	ADAM15	PD	−0.14	0.04	2.53E‐04	2.41E‐02	*CIS*	2.5E‐18
rs79671334	CTSS	Schizophrenia	0.16	0.04	1.14E‐04	1.10E‐02	*CIS*	6.15E‐05
rs3735172	RARRES2	Years of schooling	−0.02	0.005	9.29E‐05	8.92E‐03	*CIS*	0.82

To test the evidence for sharing genetic effects between pQTLs and brain‐related disorders a combination of Mendelian randomization (MR) and genetic colocalization analysis was performed. For the MR, Wald ratio (WR) tests based on the independent *cis*‐pQTL instruments available for each protein was performed. AD, Alzheimer's disease. PD, Parkinson's disease. More details are given in Dataset EV11.

**Table 2:** Candidate drugs in clinical stages for pathways identified in Mendelian randomization experiments.

Target protein	Drug name	Highest stage	Originator	Indication	Significant MR outcome
ApoE	LX1001	Phase I	LEXEO Therapeutics	AD	AD
CD33	BI‐836858	Phase II	Boehringer Ingelheim	AD	AD
GRN	AL‐001	Phase III	Alector, GSK	ALS, FTD	AD
MMP10	NA				AD (preclinical)
SIGLEC9	NA				ALS
CTCF	NA				Depression, bipolar disorder
FLRT2	NA				Focal epilepsy
RARRES2	NA				Focal epilepsy
CD38	Daratumumab	Launched	Janssen	Myeloma	PD
GPNMB	NA				PD
ADAM15	pORT‐RDD	Phase I/II	BioAlliance Pharma	Melanoma	PD
CTSS	RWJ‐445380	Phase II	Johnson & Johnson	RA	Schizophrenia

For each protein that showed nominal evidence of causality in the *cis*‐pQTL‐based Mendelian randomization analyses, we queried the Cortellis database (in September 2022) to identify clinical stage drugs targeting the identified proteins. AD, Alzheimer's disease; ALS, amyotrophic lateral sclerosis; FTD, frontotemporal lobe dementia; MR, Mendelian randomization; PD, Parkinson's disease; RA, Rheumatoid arthritis.

For the AD, preclinical AD, and PD findings, we had the opportunity to test for observational differences between diagnostic groups in CSF biomarker levels for the proteins implicated in the MR analysis (AD: APOE, CD33 and GRN; Preclinical AD: MMP‐10; PD: CD38, GPNMB, ADAM15). We found significant effects for GRN in AD, for MMP‐10 in preclinical AD, and for CD38 and GPNMB in PD, supporting the MR findings (Table EV2).

We directly compared our MR findings with the findings in Yang *et al* (2021) which included MR findings for CSF pQTLs and the outcomes AD, PD, ALS, stroke, and FTD. Among our gene‐level findings, the causal association between *CD33* (also known as *Siglec‐3*) and AD was replicated between the studies. The other MR findings were unique to our study and to those reported by Yang *et al* (2021), respectively.

### Colocalization analysis

We used the *coloc* package (Giambartolomei *et al*, 2014) to test shared causal SNPs between proteins and conditions (Table 1). Evidence of colocalization confirms that the WR effect observed is not due to the pQTL and outcome sharing genetic effects coincidentally due to LD patterns in the region. Out of the 18 pQTL‐protein pairs we found six pQTLs showing colocalization with eight different outcomes at a posterior probability > 0.75. The APOE pQTL (rs429358) had a colocalization posterior probability of 1 with AD, 0.81 with preclinical AD, 0.99 with amygdala volume, 0.87 with hippocampus volume and 0.95 with nucleus accumbens volume. CD33 pQTL (rs273634) and GRN pQTL (rs5848) had a posterior probability of 0.78 and 0.99, respectively, with AD. The SIGLEC9‐rs2258983 protein‐pQTL association had a posterior probability of 0.93 with ALS. The colocalization posterior probability for GPNMB‐rs199348 with PD was 0.93. RARRES2‐rs3735172 had a posterior probability of 0.82 with years of schooling.

### The GMNC‐OSTN region and CSF trans‐pQTLs

As noted above, intergenic variants on chromosome 3 were common among the CSF *trans*‐pQTLs. Eight genetic variants had significant *trans*‐pQTLs (*N* = 36 in total, Dataset EV12) in a region where the closest encoding gene downstream was *GMNC* (Ensembl gene ID ENSG00000205835) and the closest encoding gene upstream was *OSTN* (Ensembl gene ID ENSG00000188729). There were no plasma pQTLs in this region, emphasizing its specificity for the CSF proteome in this study. *OSTN* encodes a hormone (osteocrin) that regulates dendritic growth in cerebral cortex (Ataman *et al*, 2016), with enhanced expression in the cerebral cortex (according to The Human Protein Atlas). *GMNC* (geminin coiled‐coil domain‐containing protein‐1) is a regulator of DNA replication, with only weak expression in brain. Based on this, we suggest that *OSTN* is a possible causal gene for the CSF *trans*‐pQTLs in this region. We queried the EBI GWAS catalog (September 2022) for previous associations with variants mapped to *GMNC* or *OSTN*. There were 77 hits in total (Dataset EV13), including 42 unique genetic variants. Twenty out of 29 unique reported traits were brain‐related, mainly comprising volumetric brain measures, including of ventricle volume or white matter lesions, supporting the relevance of this genomic region for CNS‐related molecular pathways. Of the eight *GMNC‐OSTN*‐related variants that we identified, there were three cases (rs201663722, rs4308324, and rs71635338) with exact matches to variants previously described to be associated with brain volumetric measures.

### The importance of ventricle volume for CSF pQTLs

Given the importance of the *GMNC‐OSTN* region for CSF *trans*‐pQTLs and previous links between genetic variants in this region and brain ventricle volume (Vojinovic *et al*, 2018; Zhao *et al*, 2019), we hypothesized that brain ventricle volume may be a confounder for CSF pQTLs (due to dilution effects on proteins). To our knowledge, this has not been tested before. To investigate this, we utilized available magnetic resonance imaging (MRI) data in a subset of participants (*N* = 305 cognitively normal controls, *N* = 180 individuals with SCD, and *N* = 235 patients with MCI, total *N* = 720).

We first tested if the most common CSF *trans*‐pQTL variant in our study (rs71635338) was associated with ventricle volume (standardized and used as outcome in linear regression), adjusting for intracranial volume (ICV), age, sex, diagnostic group, and the top 10 principal components of the genetic background. The analysis confirmed our hypothesis that this SNP (like other previously described SNPs in the *GMNC‐OSTN* region) was associated with ventricle volume (*β* = 1.51 standard deviations larger ventricle volume for the minor allele compared to the reference allele, *P* = 0.007). Note that this was a single test, based on an *a priori* hypothesis, whereby correction for multiple comparisons was not applicable.

**Figure 6:**
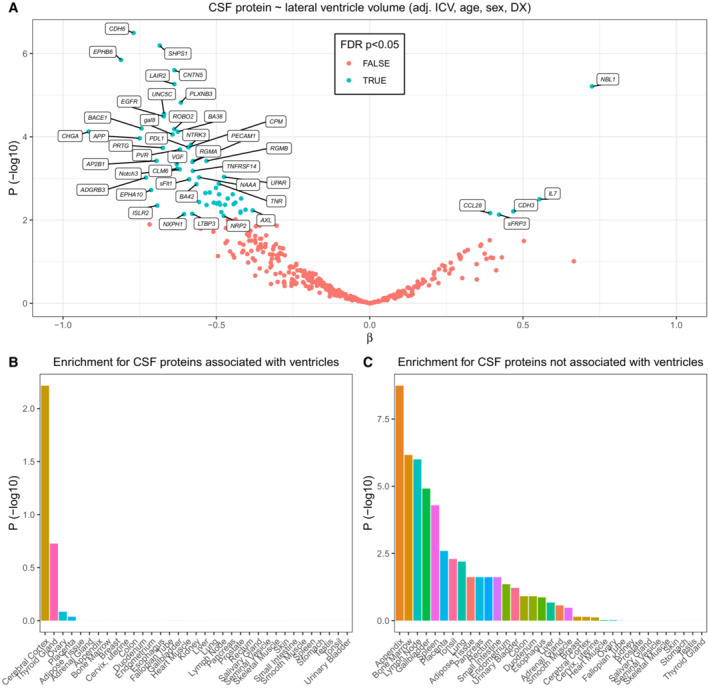
Associations between CSF proteins and ventricle volume Associations for all CSF proteins with volumes of lateral ventricles. Associations significant after multiple comparisons are indicated in blue. Test assumptions were verified by visual inspection of diagnostic plots. Selected significant proteins are annotated.Enrichment analysis for CSF proteins associated with ventricles.Enrichment analysis for CSF proteins not associated with ventricles.

We proceeded to test associations between CSF protein levels (for all CSF proteins) with lateral ventricle volume, using linear regression models with the standardized CSF protein levels as outcome variables, adjusting for ICV, age, sex, and diagnostic group. After FDR‐correction, 64 CSF proteins were associated with ventricular volume (Fig 6A). We hypothesized that CSF proteins that were associated with ventricular volume were more likely to originate in the brain (and be susceptible to dilution by CSF in the ventricles before reaching the lumbar region, where CSF is sampled through lumbar puncture), rather than the periphery (since peripheral proteins may leak into lumbar CSF through multiple pathways and may therefore be less susceptible to dilution in the ventricles). We conducted transcription‐level tissue enrichment analysis using the *TissueEnrich* package (Jain & Tuteja, 2019). Genes for proteins that were associated with ventricle volume showed strong enrichment for the cerebral cortex (Fig 6B), while the remaining proteins did not show enrichment for any brain region (Fig 6C). This supported our hypothesis that CSF proteins associated with ventricle volume are likely to originate from the brain.

**Figure EV5:**
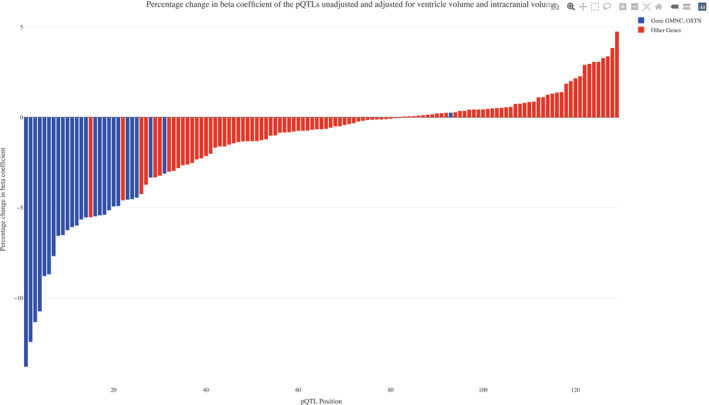
Effects on CSF pQTL from adjustment for ventricle volume This figure shows the change in CSF pQTL β‐coefficients with and without additional adjustment for ventricle volume and intracranial volume. pQTLs involving genetic variants in the *GMNC‐OSTN* region on chromosome 3 are highlighted in blue. Note that these analyses were done on a subset of participants with MRI data. The plot (and corresponding Dataset EV14) is restricted to CSF protein‐pQTL pairs that were at least genome‐wide significant in the full cohort and at least significant with *P* < 5 × 10^−6^ in the MRI subcohort. Full interactive functionality is provided in the Source Data file “Fig EV5 Percent_Change_beta_ICV_VV.html”, which is available online. Source data are available online for this figure.

Finally, we tested if the pQTL residuals (from the main pQTL analysis, using all CSF pQTLs) were associated with ventricle volume. When adjusting for ventricle volume (and ICV), the β‐coefficients were changed by up to 14% (*β*‐coefficients were generally reduced, while *P*‐values were generally increased) (Fig EV5). These results support ventricle volume as a confounder for some CSF pQTL analyses. In particular, we noted that several CSF protein‐pQTL pairs with effects of adjustment from ventricular volume involved the *GMNC‐OSTN* region on chromosome 3. For example, this region was implicated in 19 of the 20 CSF pQTLs with largest reductions in β‐coefficients following ventricle adjustment, further implicating this genomic region in the interplay between genetic regulation, brain anatomy, and the CSF proteome.

## Discussion

In this study, we provide a comprehensive genome‐wide analysis of associations between genetic variants and a large number of CSF proteins with the aim to clarify the genetic regulation of the CSF proteome, an issue that has received only limited attention in the past (Sasayama *et al*, 2017; Yang *et al*, 2021). We identified several genetic variants that were associated with protein levels in the CSF, which may have implications for protein regulation in the central nervous system. Comparisons against previously published eQTLs showed that several CSF pQTLs correlated with gene expression in the brain, supporting their biological relevance. Comparisons against plasma pQTLs on the same subjects support that some proteins have distinct genetic regulation within the central nervous system that is different from that in the periphery. A genomic region on chromosome 3, shown before to be related to brain morphology, was implicated for several CSF‐specific pQTLs. Using MR analyses on *cis*‐pQTL findings, we identified CSF proteins that were implicated in different neurological diseases, including AD, PD, ALS, depression, bipolar disease, and epilepsy, as well as brain volume measures. We also demonstrate that CSF pQTLs may be susceptible to confounding effects of brain ventricular volume.

Several CSF proteins identified in the MR analysis for AD (GRN), preclinical AD (MMP‐10), and PD (CD38 and GMBP) were also validated with observational differences between patients and controls. We did not find altered levels for CD33 or APOE (for AD) or ADAM15 (for PD). Hypothetically, this may be disease‐stage dependent, since causal biomarkers may not always show cross‐sectional differences during the later stages of the disease. We note that another recent CSF pQTL study also implicated CD33 for AD by MR analysis (Yang *et al*, 2021), while several other proteins were uniquely implicated in this study versus in Yang *et al* (2021). The proteins identified in the MR analyses may be relevant to pursue as new treatment targets, and we note that candidate treatments are in development for some of these already. In Table 2, we list available drug candidates (including proposed for non‐neurological indications), which could potentially be explored for the neurological diseases identified in this study. Notably, several findings from our MR analysis are not listed already as drug targets and may be particularly interesting to explore as new targets. One particularly interesting candidate may be GPNMB in PD. GPNMB (Glycoprotein nonmetastatic melanoma protein B) is a transmembrane glycoprotein, which has been studied before in cancer and inflammation (Saade *et al*, 2021). Its potential role in PD pathogenesis and links to aggregation of α‐synuclein has recently been highlighted (Diaz‐Ortiz *et al*, 2022). Regarding the role of MMP‐10 (matrix metalloproteinase 10) in preclinical AD, we note that previous studies found increased levels of CSF MMP‐10 in AD, as well as increased rates of disease progression in those with high CSF MMP‐10 levels at baseline (Martino Adami *et al*, 2022). Regarding the MR findings of CD33 (SIGLEC‐3) in AD and SIGLEC‐9 in ALS, we note that several members of the SIGLEC protein family have been implicated for roles in neurodegenerative diseases, especially due to neuroinflammatory properties (Siddiqui *et al*, 2019). The MR analyses also implicated FLRT2 and RARRES2 in epilepsy. To our knowledge, FLRT2 (fibronectin leucine‐rich transmembrane protein 2) has not been described extensively in relation to epilepsy or seizures, but there is evidence that this cell adhesion molecule is involved in neuronal development, including development of inhibitory cortical circuits (Fleitas *et al*, 2021). RARRES2 (retinoic acid receptor responder protein 2, also known as chemerin) is described as having chemotactic properties during inflammatory responses and serum RARRES2 levels were associated with severity of seizures in children with idiopathic epilepsy (Elhady *et al*, 2018). CTSF (cathepsin F), implicated here for depression and bipolar disease, is a lysosomal protein which is involved in the pathogensis of some types of neuronal ceroid lipofuscinosis (Smith *et al*, 2013).

In a novel approach which integrated volumetric MRI, we found that several genetic variants and CSF proteins were associated with volumes of the cerebral ventricles. Previous studies have demonstrated associations between individual CSF biomarkers (for the AD biomarkers Aβ, T‐tau, and P‐tau) and ventricle volume (likely dilution effects; Edsbagge *et al*, 2017; van Waalwijk van Doorn *et al*, 2017), but to our knowledge, this is the first large‐scale study of such associations, and how they affect associations between CSF biomarker and genetic variants. The results point to ventricle volume as a possible confounder for many CSF pQTL studies, although we demonstrate that most findings remain significant (but sometimes attenuated) after adjustment for ventricle volume. Recently, it was reported that genetic variants in the *GMNC‐OSTN* region on chromosome 3 (described in detail in this study) were associated both with smaller ventricles and with increased CSF levels of the AD biomarker P‐tau (Jansen *et al*, 2022). This finding may first be perceived as counterintuitive, since smaller ventricles is generally thought to indicate less neurodegeneration, and increased CSF P‐tau is generally associated with more neurodegeneration. However, the findings in our current study suggest that the association described by Jansen *et al* (2022) may have been confounded by the genetic effects on brain anatomy, causing differences in dilution and concentration across a range of CSF biomarkers. We noted that variants in the *GMNC‐OSTN* region were common among the CSF pQTLs where ventricular volume had large confounding effects (Fig EV5). Future studies of the genetic regulation of the CSF proteome may therefore benefit from an integration of brain imaging, as demonstrated here, to adjust for such confounding effects.

The overall genetic architecture of the CSF pQTLs was similar to findings reported for plasma pQTLs (Sun *et al*, 2018), gene expression QTLs (Stranger *et al*, 2012), and a recent study on CSF pQTLs (Yang *et al*, 2021). As shown in our detailed EBI GWAS query, most of the findings for CSF pQTLs were completely or partly novel. In particular, our study (comprising 1,591 individuals using mainly PEA and mass spectrometry assays for 398 proteins) and the study by Yang *et al* (2021) (comprising 971 individuals, using aptamer technology with 1,305 aptamers) provide complementary evidence. Yang *et al* (2021) described 226 CSF *cis*‐pQTLs (at *P* < 5 × 10^−8^) and 48 *trans*‐pQTLs (at Bonferroni corrected *P*) compared to 158 CSF *cis*‐pQTLs (at *P* < 5 × 10^−8^) and 59 CSF *trans*‐pQTLs (at Bonferroni corrected *P*) in our study. A direct comparison between the results showed that 15 pQTLs were exactly replicated between the studies (the same variant and protein and with the same direction of effect size with respect to risk allele). The overall replication rate between the studies was therefore quite low. This suggests that although some pQTLs can be robustly replicated across distinct proteomics methods, aptamer‐ and antibody‐based technologies largely provide non‐overlapping information. A recent large‐scale study directly compared the aptamer‐based SomaScan technology with antibody‐based PEA and found considerable differences in results (Katz *et al*, 2022), which is in accordance with our findings. Importantly, Katz *et al* (2022) found evidence supporting more reliable protein target specificity and a higher number of phenotypic associations for the PEA assays, used in the current study. The specificity of aptamer‐based multiplex methods has also been discussed before (Christiansson *et al*, 2014; Joshi & Mayr, 2018). Applied together, these technologies may help to increase our understanding of the genetic regulation of the CSF proteome. Another possibility is that the differences between results in our study and Yang *et al* (2021) could partly also be cohort dependent.

In a comparison between CSF pQTLs and brain eQTLs, we noted that there were both concordant associations, supporting direct links between gene transcription and translation, as well as discordant associations. Discordant associations between pQTLs and eQTLs have been reported to varying degrees before (preprint: Sun *et al*, 2022) and may hypothetically reflect biological pathways with negative feedback loops that regulate protein levels. As recently pointed out, eQTL and disease‐associated loci may have fundamental differences, contributing to different results with these approaches (preprint: Mostafavi *et al*, 2022).

Our study is not without limitations. Our cohort consisted of elderly people, which limits the generalizability of the results to younger populations. We predominantly used PEA for protein quantification, which rely on matched pairs of antibodies linked to unique oligonucleotides. Although this is a very sensitive method for protein quantification, it is possible that missense variants could affect the binding of the assay antibodies to the target protein. This could hypothetically result in an erroneous pQTL determination (i.e., if the variant significantly reduces binding of the antibody to the protein, the result may erroneously be interpreted as a pQTL negatively regulating protein levels). We cannot exclude that the MR analysis may be affected by “pseudo pQTLs” or pQTLs driven by epitope effects, i.e., that the assay detects protein with missense variant differently (especially for *APOE*, *SIGLEC9*, and *FLRT2*, which were missense variants). Mass spectrometry‐based methods, which simultaneously measure across multiple peptides per protein, may be less susceptible to this type of artifact (Solomon *et al*, 2018), and were used in this study for quantification of certain proteins (Dataset EV1). Furthermore, the genetic association to a protein does not necessarily give information about the actual functional consequence of a genetic variant (i.e., the variant might alter total expression, or expression of one particular isoform of the protein that is detected, but not others). Another limitation is that although we studied ~ 400 proteins, this is only ~ 2% of the proteome. Future studies using the most recently developed proximity extension assays may extend this to closer to 3,000 proteins. Due to lack of availability of post‐mortem brain tissue, our study was limited to protein quantification in CSF and blood plasma. As proteomics technologies develop to facilitate more sensitive measures in bulk brain tissue, this will allow us to assess the overlap more accurately between protein expression in brain tissue and spinal fluid. More comprehensive genetic characterization, via whole exome or whole genome sequencing, is recommended to address possible artifacts in future studies.

To conclude, we provide a comprehensive overview of the genetic regulation of the CSF proteome, which adds to the previous literature in this field and provides new tools to study the central nervous system in normal physiological conditions and disease. The main novelties included the use of highly specific proximity extension assays in a large cohort, and the integration of brain volumetrics to account for confounding factors. New possible treatment targets for several neurological diseases were nominated.

## Material and Methods

### Participants

Participants were recruited in the prospective and longitudinal BioFINDER study (NCT01208675) from 2009 to 2014, at three neurology and memory clinics in southern Sweden. The participants included CU participants (recruited as cognitively normal controls or as SCD subjects), patients with MCI, patients with dementia due to AD, FTD, DLB, PDD, VaD, or unspecified dementia. We also included patients with PD, MSA, CBS, PSP, or unspecified parkinsonism. Details on recruitment were presented before (Hall *et al*, 2012; Mattsson *et al*, 2016). In short, cognitively normal participants were included if they (i) were aged 60–80 years, (ii) had Mini Mental State Examination (MMSE) scores of 28–30 at their initial screening visit, (iii) lacked symptoms of cognitive impairment, as assessed by a physician, and (iv) did not fulfill the criteria for MCI or dementia. Participants were excluded from the control group if they had a significant neurological or psychiatric disease, current alcohol or substance misuse. SCD and MCI patients were included if they (i) were aged 60–80 years, (ii) had complaints related to memory, executive, visuo‐spatial, language praxis, or psychomotor function, (iii) had MMSE scores of 24–30, (iv) had essentially preserved activities of daily living, and (v) (MCI only) had significant impairment in at least one cognitive domain according to an assessment by an experienced neuropsychologist. Exclusion criteria for SCD and MCI included fulfillment of criteria for any dementia disorder, or cognitive impairment that could be definitively explained by another condition. AD dementia patients were classified using the criteria for probable AD, as defined by National Institute of Neurological and Communicative Disorders and Stroke–Alzheimer's Disease and Related Disorders Association (McKhann *et al*, 1984). PD patients met the National Institute of Neurological Disorders and Stroke diagnostic criteria for PD (Gelb *et al*, 1999). PDD patients met the clinical diagnostic criteria for dementia associated with PD according to Emre *et al* (2007). MSA patients met the Gilman *et al* (1999) consensus statement on MSA diagnosis (Gilman *et al*, 1999). PSP patients met the National Institute of Neurological Disorders and Stroke–Society for Progressive Supranuclear Palsy International Workshop criteria (Litvan *et al*, 1996a). CBD patients were diagnosed in accordance with the guidelines by Litvan *et al* (1996b). DLB patients met the consensus criteria according to McKeith *et al* (2005). General exclusion criteria in all groups included systematic illness preventing participating in the study, and refusal to undergo lumbar puncture. All BioFINDER participants with available CSF biomarker data and genotyping data were included. The BioFINDER study was approved by the regional Ethical Review Board in Lund (2008/289, 2008/695, 2010/156, 2010/401, 2013/494). All participants provided informed consent and the study conformed to the principles set out in the WMA Declaration of Helsinki and the Department of Health and Human Services Belmont Report.

### Sampling

CSF and plasma samples were collected and analyzed following a standardized protocol (Palmqvist *et al*, 2017). Samples were centrifuged (2,000 *g*, +4°C, 10 min), aliquoted into 1 ml polypropylene tubes (Sarstedt AG & Co., Nümbrecht, Germany), and stored at −80°C. Before protein analyses, all samples underwent one freeze–thaw cycle and were aliquoted into 200uL LoBind tubes (Eppendorf Nordic A/S, Denmark).

### Biochemical analyses

Most proteins were measured using validated, highly sensitive and specific PEA developed by Olink Proteomics (Uppsala, Sweden) as described before (Whelan *et al*, 2019). In short, four commercially available Proseek® Multiplex panels (CVD‐III, INF‐I, NEU‐I, NEU‐EXP) were used to measure the concentrations of proteins in CSF and plasma, following the manufacturer's protocol (Assarsson *et al*, 2014). Each panel had 92 proteins, and 6 proteins were included in two panels, giving a total of 362 unique proteins. Proteins with < 50% of values below the reported lower limit of quantification (LLQ) were analyzed quantitatively. For proteins with > 50% of values below the LLQ (25 proteins in plasma and 128 in CSF), we used the actual raw extended values, provided by Olink, to impute best‐guess values. All proteins passed quality control (QC) (with warnings indicated in < 20% of samples) in all measured samples, except for BDNF and CCL22, which were therefore removed from all analyses (note that these proteins were later highlighted by Olink Proteomics as having technical issues and were replaced with other proteins in subsequent versions of the assays). Quantities for the 360 remaining proteins were used as normalized protein expression (NPX) values on the log_2_ scale. Intra‐ and inter‐plate coefficients of variation (CV) of the panels are available from Olink Proteomics AB.

In addition to Olink Proteomics proteins, we also analyzed additional proteins using other assays, including ELISAs (for CSF and plasma Aβ38, BACE1, Hb, neurogranin, neurofilament heavy chain, and CSF Aβ40, Aβ42, P‐tau181, T‐tau, EUROIMMUN), Meso Scale Discovery (for CSF a‐synuclein, bFGF, CRP, sICAM1, IL1223p40, IL16, IL7, PlGF, SAA, sFLT1, sTREM2, sVCAM1, VEGF‐D), mass spectrometry‐based assays (for CSF AP2B1, ApoE, APP, CHGA, CTSF, GM2A, NPTX1, SCG2, SNAP25, SYT1, Ubiquitin, VGF, Tau224), Elecsys assays (for plasma Aβ40 and Aβ42) and immunoturbidimetry (CSF and plasma albumin). The non‐Olink proteins all passed thresholds of being quantifiable and passing QC in at least 80% of measured samples.

The laboratory staff analyzing samples were blinded to all data. Assay batches were randomized with respect to covariates. There was no significant variance in findings related to assay batches. Taken together, we analyzed 398 proteins in CSF and 368 in plasma (see Dataset EV1 for overview).

### Genomic data

Genotyping was conducted using the Illumina platform GSA‐MDA v2. Subject‐level QC was conducted, including the removal of sexual incompatibility between chip‐inferred sex and self‐reported sex, low call rates (5% cut‐off), and extreme heterozygosity. Relatedness among the samples was eliminated by removing one participant from each pair of close relatives (first or second degree) identified as $\hat{\pi }$ ≥ 0.0625. Multi‐dimensional scaling was done using PLINK2 to create principal components in genetic analyses to account for ancestry. Standard QC steps were performed for SNP‐level filter to ensure conformity with the reference panel used for imputation (strand continuity, names of the alleles, position and assignments for Ref/Alt).

For imputation, 685,494 high‐quality variants (autosomal, bi‐allelic variants with Hardy–Weinberg Equilibrium (HWE) *P* > 5 × 10^−8^ and with a call rate of > 99%) were used. Imputation was carried out using the Michigan Imputation Server (link) with SHAPEIT for phasing, Positional Burrows‐Wheeler Transform (PWBT) for imputation and the entire Haplotype Reference Consortium (release 1.1) reference panel. Multi‐allelic variants and SNPs with a data imputation score < 0.2 were excluded as part of post‐imputation QC and genotype calls with a posterior likelihood < 0.9 were set to fail (i.e., hard‐called). SNPs with a genotyping rate > 0.9 were retained. SNPs with MAF ≥ 1% were used for the analyses.

### Identification of pQTLs

For each protein, a linear regression analysis was performed with adjustments for age, gender, diagnostic group, collection site, and time from sampling to assay execution. The residuals from the regressions were standardized and transformed using rank‐based inverse‐normal transformation (Sun *et al*, 2018). The genome‐wide association analysis to identify pQTLs were performed using PLINK1.9 with adjustment for genetic principal components to adjust for population stratification. This use of covariate adjustment follows standard procedures (Folkersen *et al*, 2020) in pQTL analyses. Linkage disequilibrium (LD) analysis was performed to select the lead SNP within the LD region, using a correlation of R2 = 0.1 to identify independent genetic loci.

### Annotation of pQTLs

pQTLs were defined as *cis*‐pQTLs when the genomic location of the genetic variant was proximal (< 1 Mbp) to the protein‐coding gene, and *trans*‐pQTLs when the genetic variant was more distant (Fauman & Hyde, 2022). All genes within 1 Mbp up‐ or downstream from the variant with the lowest *P*‐value for protein level association were annotated. The distance between the pQTL and gene was calculated for each gene in the gene set. The best guessed gene for the pQTL was determined from each of these gene sets as the shortest distance between the pQTL and gene minimum among all the calculated distances, following previously described methods (Stacey *et al*, 2019; Nasser *et al*, 2021). Enrichment of functional consequences of lead (and SNPs in LD) pQTLs, defined as genome‐wide significant, was performed using the ANNOVAR enrichment test (Wang *et al*, 2010) from FUMA (Watanabe *et al*, 2017, 2019).

### GWAS catalog comparisons

In order to assess the relevance and novelty of our pQTLs, we compared all our lead SNPs to all the lead SNPs reported in the GWAS catalog. We downloaded all the lead SNPs from the GWAS catalog (https://www.ebi.ac.uk/gwas/api/search/downloads/alternative). We retained all entries that were within 250 Kbp of any of our lead SNPs. For each of our lead SNPs, we recorded (i) the total number of GWAS catalog entries, (ii) the total number of pQTLs, (iii) the total number of distinct protein biomarkers, (iv) the total number of “CNS disease” traits, and (v) the total number of distinct “CNS disease” traits within 250 Kbp of that lead SNP. A GWAS association was defined as representing a “CNS disease” trait if one or more of the Experimental Factor Ontology terms assigned by the GWAS catalog curators was a descendant of the term “central nervous system disease” in that ontology (http://www.ebi.ac.uk/efo/EFO_0009386). There are 2,717 descendants of “CNS disease” although certainly they are not all represented in the GWAS catalog.

GWAS associations in the GWAS catalog were defined as representing pQTLs through a laborious manual review of all 300,000+ GWAS catalog associations. An association was defined as a pQTL if the trait represents the abundance of the protein product of a human gene. Each association defined as a pQTL was tagged with the human gene or genes encoding the protein (or subunits of the protein if it is a heteromultimer, such as hemoglobin). While most published pQTLs represent protein abundance from plasma or serum samples, a small number represent prior CSF pQTL studies. These were identified by the presence of the word “CEREBROSPINAL” in the “DISEASE/TRAIT” field in the GWAS catalog. Novelty of the CSF pQTLs identified in the current study was assessed on multiple levels, as described in the Results.

### Mendelian randomization

To test whether the CSF proteins were likely to play a causal role in neurological disease, we performed 2‐sample MR analysis using *cis*‐pQTLs to model exposure and genetic case/control studies different traits as outcomes (see Results for details). We used the *TwoSampleMR* R package (Hemani *et al*, 2018) with the CSF pQTL variants identified in the current study (at Bonferroni corrected *P*‐value < 1.253 × 10^−10^) as IVs.

The outcome data came from several sources and included GWAS summary statistics data for genetic risks for clinical AD (Kunkle *et al*, 2019), PD (Chang *et al*, 2017), FTD (Ferrari *et al*, 2014), and amyotrophic lateral sclerosis (ALS; Nicolas *et al*, 2018). For preclinical AD, we performed an in‐house GWAS analysis using data from the A4 study (Insel *et al*, 2020). Genetic data from A4 underwent a quality control process limiting to variants with a call rate > 95%, minor allele frequency > 1%, if cryptic relatedness was identified, or if out of Hardy–Weinberg equilibrium (*P* > 10^−6^). Imputation was performed using the European samples from the HRCr1.1.2016 reference panel (Build 37 Assembly 19). Aβ PET imaging in A4 was done using 18F‐florbetapir data, acquired 50–70 min post‐injection. Images were realigned and averaged, and then spatially aligned to a standard space template. 18F‐florbetapir, sampled in a global neocortical region for Aβ, was expressed as an SUVR with a cerebellar reference region. 18F‐florbetapir PET SUVRs were regressed on each genetic variant separately, adjusting for age, sex, and the first five principal components of the genetic background to account for unmeasured population stratification. SUVRs were modeled using ordinary least squares regression assuming a linear association with allele frequency. Statistical significance of genetic variants was determined using Wald tests and adjusted using FDR correction.

Prior to downstream analyses, we checked and verified that there was no allelic heterogeneity or locus heterogeneity in the available GWAS summary statistics files. Where a single variant was used as the IV, we performed a Wald ratio (WR) test. In the rare cases where there were multiple genetic variants as IV, we used the fixed effects inverse variance‐weighted (IVW) method. Estimates of the causal effect of proteins on disease were interpreted as per standard error increments in inverse‐rank normalized protein level.

### Colocalization analysis

To estimate the prior likelihood of each genomic locus having a single variant affecting both the protein and the phenotype, we used a strict Bayesian model (colocalization analysis) using the *coloc* package in R (Giambartolomei *et al*, 2014). This method estimates the following five cases (i) neither trait has a genetic association in the region (H_0_); (ii) only trait 1 has a genetic association in the region (H_1_); (iii) only trait 2 has a genetic association in the region (H_2_); (iv) both traits are associated, but with different causal variants (H_3_); (v) both traits are associated and share a single causal variant (H_4_). It also includes specifying a prior likelihood for a SNP being correlated with disease only (p_1_), protein level only (p_2_), and with both traits (p_12_). With p1 and p2 set to 1 × 10^−4^, we applied the default *P* values, assuming that 1 in 10,000 SNPs is causal for either trait and p12 was set to 1 × 10^−5^.

**The paper explained**

**Problem**

Many brain diseases lack efficient disease‐modifying treatments. Studies of the genetic regulation of cerebrospinal fluid (CSF) proteins can give clues to novel targetable biological pathways of brain diseases. However, only a few studies have combined genotyping data and CSF data on large sets of proteins, which is needed for this type of analysis. There is especially a lack of studies using sensitive and specific antibody‐based methods, such as proximity extension assays, for a large number of proteins.

**Results**

We used a well‐phenotyped cohort with 1,591 study participants, who had CSF biomarker data for 398 proteins and genotyping using a comprehensive array. We identified 176 significant (after Bonferroni correction) protein quantitative trait loci (pQTL), which were associated with altered CSF protein levels. In Mendelian randomization experiments, which also leveraged genetic risk data for traits related to brain diseases, we identified proteins with shared genetic effects with several diseases, including Alzheimer's disease, Parkinson's disease, amyotrophic lateral sclerosis, and others. We also identified ventricular volume as a possible confounder for CSF pQTL studies.

**Impact**

This study provides a comprehensive overview of the genetic regulation of a large number of CSF proteins, analyzed by sensitive and specific assays. These findings can be used to better understand the unique regulation of the CSF proteome in health and disease. Key proteins that were identified with shared genetic effects with brain diseases can be explored as targets for novel treatments against neurological diseases.

#### Description of supplemental data

The supplemental information includes details and summaries of pQTL findings, between‐assays comparisons for CSF biomarkers, comparisons with other CSF pQTL studies, possible gene–protein interactions for *trans*‐pQTLs, effects on CSF pQTLs from adjustment for ventricle volume, correlations between pQTLs in plasma and CSF, comparisons with eQTL findings, and summaries of Mendelian randomization results.

## Author contributions

**Oskar Hansson:** Conceptualization; resources; supervision; funding acquisition; investigation; methodology; project administration; writing—review and editing. **Atul Kumar:** Data curation; software; formal analysis; visualization; methodology; writing—review and editing. **Shorena Janelidze:** Data curation; formal analysis; investigation; methodology; writing—review and editing. **Erik Stomrud:** Methodology; project administration; writing—review and editing. **Philip S Insel:** Formal analysis; investigation; methodology; writing—review and editing. **Kaj Blennow:** Resources; methodology; writing—review and editing. **Henrik Zetterberg:** Resources; methodology; writing—review and editing. **Eric B Fauman:** Data curation; investigation; methodology; writing—review and editing. **Åsa K Hedman:** Investigation; methodology; writing—review and editing. **Michael W Nagle:** Data curation; formal analysis; investigation; methodology; writing—review and editing. **Christopher D Whelan:** Investigation; methodology; writing—review and editing. **Denis Baird:** Software; formal analysis; investigation; methodology; writing—review and editing. **Anders Mälarstig:** Data curation; supervision; investigation; methodology; writing—review and editing. **Niklas Mattsson‐Carlgren:** Conceptualization; resources; data curation; formal analysis; funding acquisition; validation; investigation; visualization; methodology; writing—original draft.

## Disclosure and competing interests statement

NMC, ES, and AK have nothing to disclose. AM, ÅKH, and EF are employees of Pfizer AB. OH has acquired research support (for the institution) from AVID Radiopharmaceuticals, Biogen, Eli Lilly, Eisai, GE Healthcare, Pfizer, and Roche. In the past 2 years, he has received consultancy/speaker fees from Roche, Genentech, Siemens, Biogen, Alzpath, and Cerveau. KB has served as a consultant, at advisory boards, or at data monitoring committees for Abcam, Axon, Biogen, JOMDD/Shimadzu. Julius Clinical, Lilly, MagQu, Novartis, Pharmatrophix, Prothena, Roche Diagnostics, and Siemens Healthineers, and is a co‐founder of Brain Biomarker Solutions in Gothenburg AB (BBS), which is a part of the GU Ventures Incubator Program, all unrelated to the work presented in this paper. HZ has served at scientific advisory boards and/or as a consultant for Abbvie, Alector, Annexon, Artery Therapeutics, AZTherapies, CogRx, Denali, Eisai, Nervgen, Pinteon Therapeutics, Red Abbey Labs, Passage Bio, Roche, Samumed, Siemens Healthineers, Triplet Therapeutics, and Wave, has given lectures in symposia sponsored by Cellectricon, Fujirebio, Alzecure, Biogen, and Roche, and is a co‐founder of Brain Biomarker Solutions in Gothenburg AB (BBS), which is a part of the GU Ventures Incubator Program (outside submitted work). CDW and DB are employees of Biogen Inc. MWN is an employee of Eisai, Inc.

## For more information

- OLINK Proteomics info about CCL22 and BDNF, https://www.olink.com/bdnf-info/
- OLINK Proteomics info about assay performance, https://www.olink.com/resources-support/document-download-center/
- The GTEx project database, https://gtexportal.org/
- The Human Protein Atlas, https://www.proteinatlas.org
- The Swedish BioFINDER study, http://www.biofinder.se

## Supporting information



Appendix

Expanded View Figures PDF

Table EV1

Table EV2

Dataset EV1

Dataset EV2

Dataset EV3

Dataset EV4

Dataset EV5

Dataset EV6

Dataset EV7

Dataset EV8

Dataset EV9

Dataset EV10

Dataset EV11

Dataset EV12

Dataset EV13

Dataset EV14

Source Data for Expanded View

PDF+

## Data Availability

Anonymized data will be shared by request from a qualified academic investigator and as long as data transfer is in agreement with EU legislation on the general data protection regulation and decisions by the Ethical Review Board of Sweden and Region Skåne, which should be regulated in a material transfer agreement.
