# Supplementary material for: The genetic regulation of protein expression in cerebrospinal fluid
Source: EMBO Mol Med. 2022 Dec 12;15(1):e16359. doi: 10.15252/emmm.202216359 (PMC9832827; doi:10.15252/emmm.202216359)
Supplement: Supplementary file 1 — Appendix [file EMMM-15-e16359-s003.docx]

**Contents:**

Appendix Figure S1: pQTL findings by analytical platform and panel, page 2

Appendix Figure S2: Sensitivity analysis in healthy controls, page 3

Appendix Figure S3: Overview of plasma pQTL architecture, page 4

Appendix Figure S4: eQTLs (GTEx data) and CSF pQTLs for corresponding genetic variants and proteins, page 5

Appendix Figure S5: Gene-protein interactions for trans-pQTLs, page 6

**Appendix Figure S1. pQTL findings by analytical platform and panel**


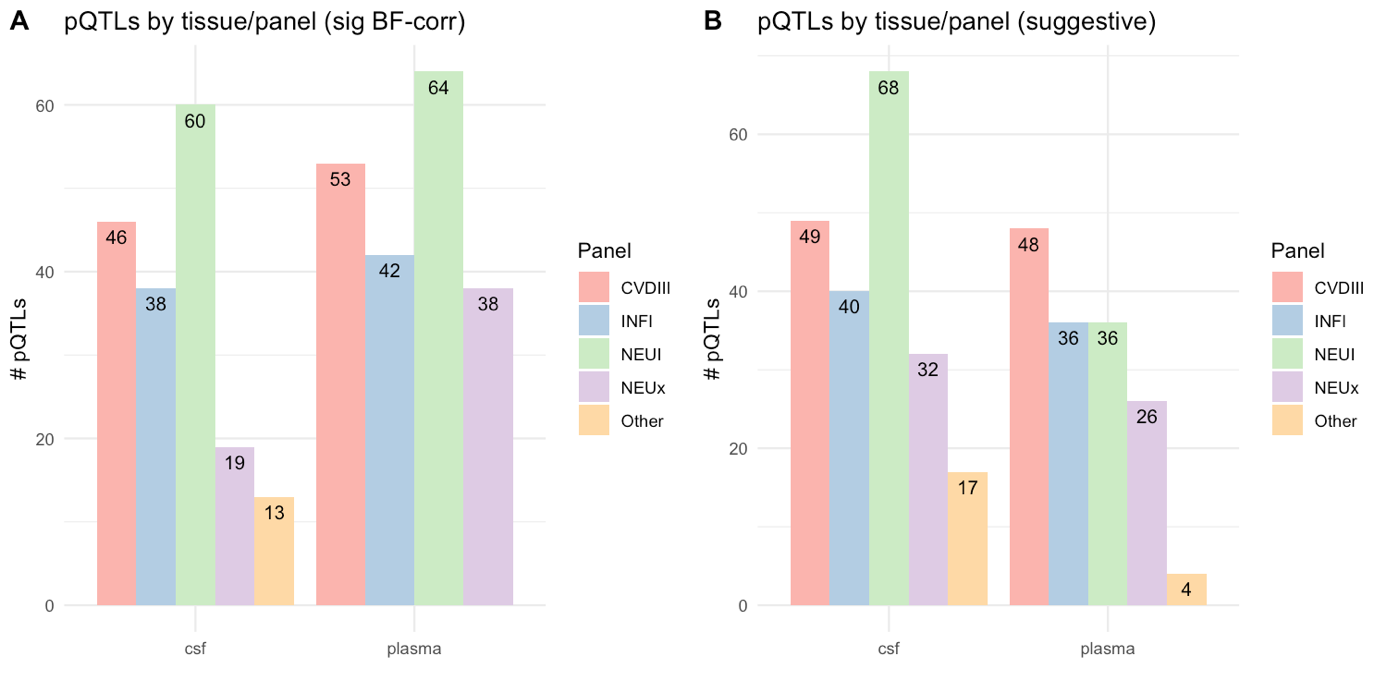


The figure shows number of pQTL significant after Bonferroni correction (panel A), or at genome-wide significance but not significant after Bonferroni correction (panel B), for CSF and plasma pQTLs. CVDIII, INFI, NEUI and NEUx are different OLINK panels.

**Appendix Figure S2. CSF pQTL sensitivity analysis in healthy controls**

The original CSF pQTL results (y-axis) versus the results when restricting the analysis to the healthy controls (x-axis). Overall, the results were similar for the vast majority of CSF pQTLs.

**Appendix Figure S3. Overview of plasma pQTL architecture**


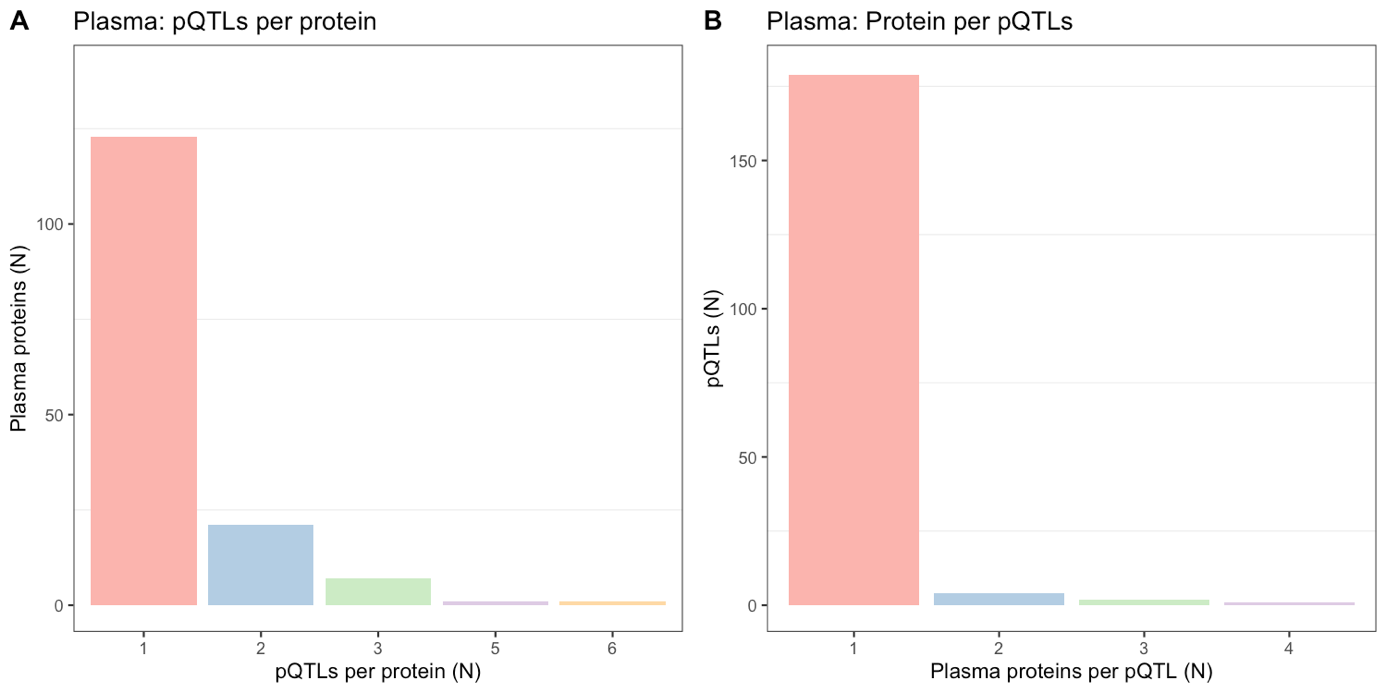


The plots show number of pQTLs per plasma protein (panel A), and number of plasma proteins per top genetic variant among the identified pQTLs (panel B).

**Appendix Figure S4. eQTLs (GTEx data) and CSF pQTLs for corresponding genetic variants and proteins**

The plot includes significant CSF pQTLs and eQTLs, from the GTEx database Most significant variants were concordant as eQTLs and pQTLs. The results largely agreed with the eQTL analysis presented in Figure 5 the main manuscript, with eQTL meta-analysis data from Sieberts et al, 2020.

**Appendix Figure S5. Gene-protein interactions for trans-pQTLs**


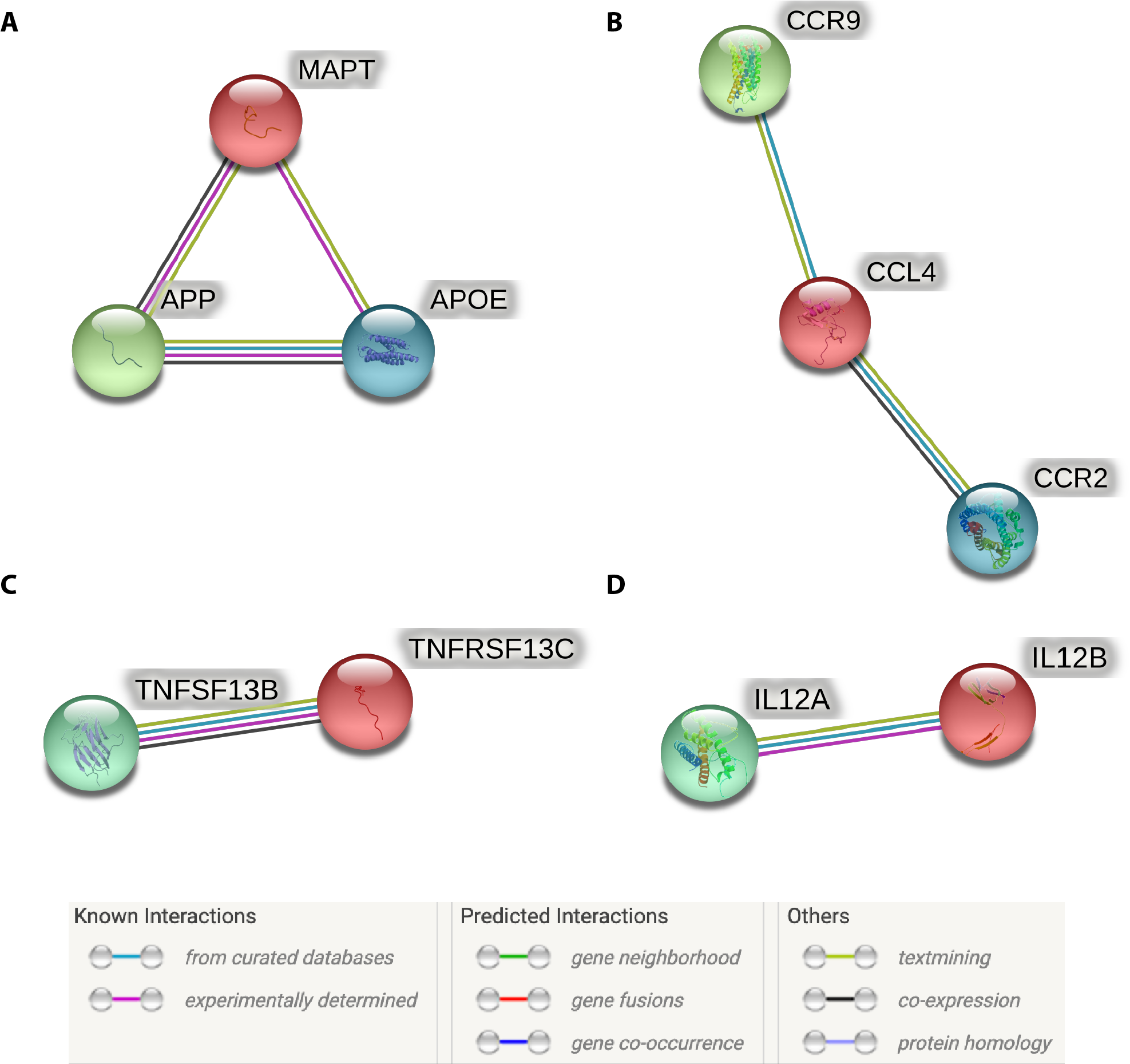


This figure shows interaction figures from the STRING/KEGG database for the CSF *trans*-pQTLs which reached an a priori determined interaction score limit (>0.7). Panel A shows *APOE* versus APP (Ab42) and MAPT (P-tau and T-tau) proteins, panel B shows CCL4 versus *CCR2* and *CCR9* genes, panel C shows TNFSF13B versus *TNFRSF13C,* and panel D shows IL12A versus *IL12B*. The plots were generated using tools available at http://string-db.org.
