# Supplementary material for: The genetic regulation of protein expression in cerebrospinal fluid
Source: EMBO Mol Med. 2022 Dec 12;15(1):e16359. doi: 10.15252/emmm.202216359 (PMC9832827; doi:10.15252/emmm.202216359)
Supplement: Supplementary file 2 — Expanded View Figures PDF [file EMMM-15-e16359-s019.pdf]

# Expanded View Figures

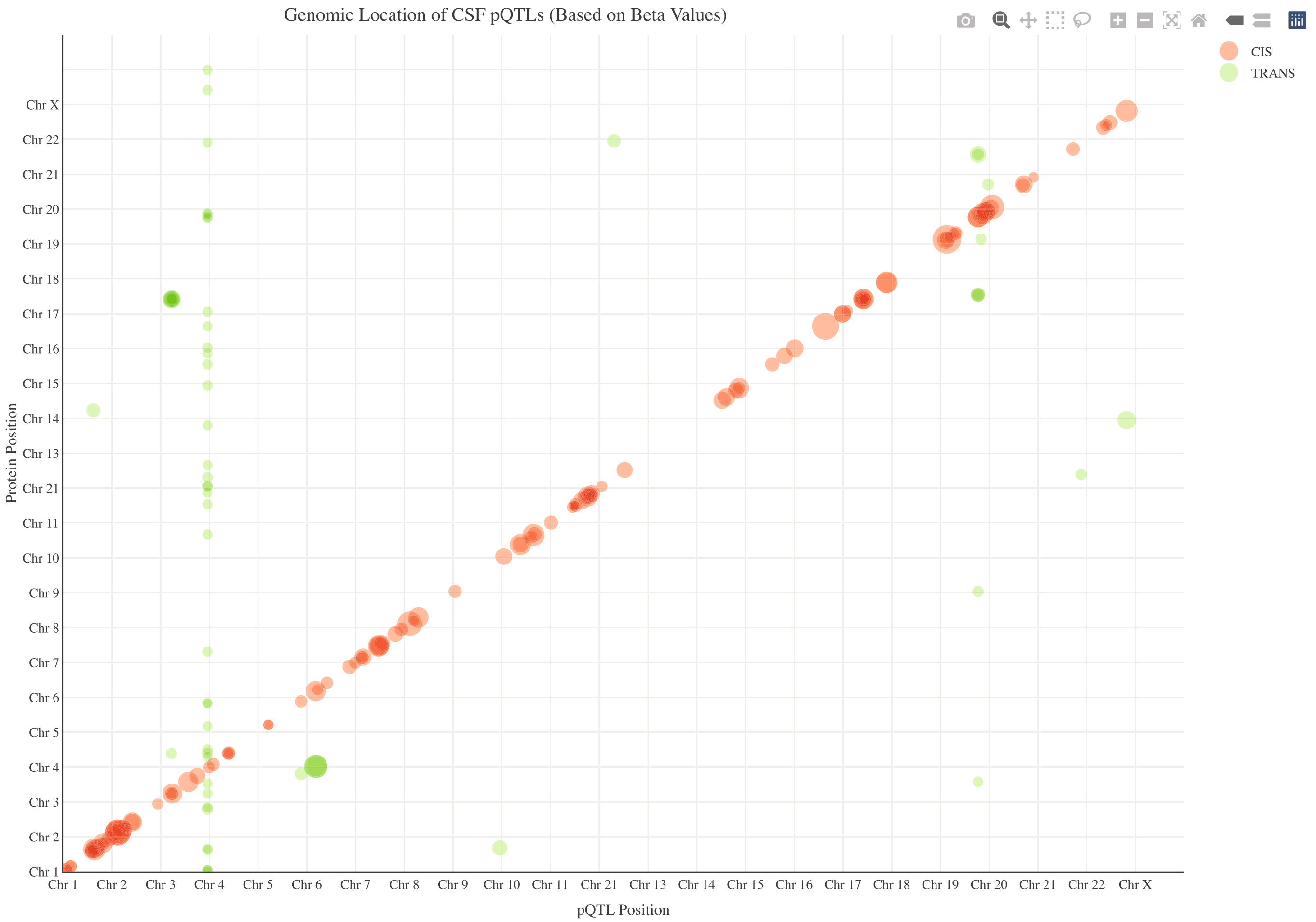


**Figure EV1. Interactive pQTL genomic map for CSF pQTLs.**

This is an interactive version of main Fig 2. Full interactive functionality is provided in the Source Data file "Fig EV1 CSF_pQTL_interactive.html", which is available online. Source data are available online for this figure.

© 2022 The Authors

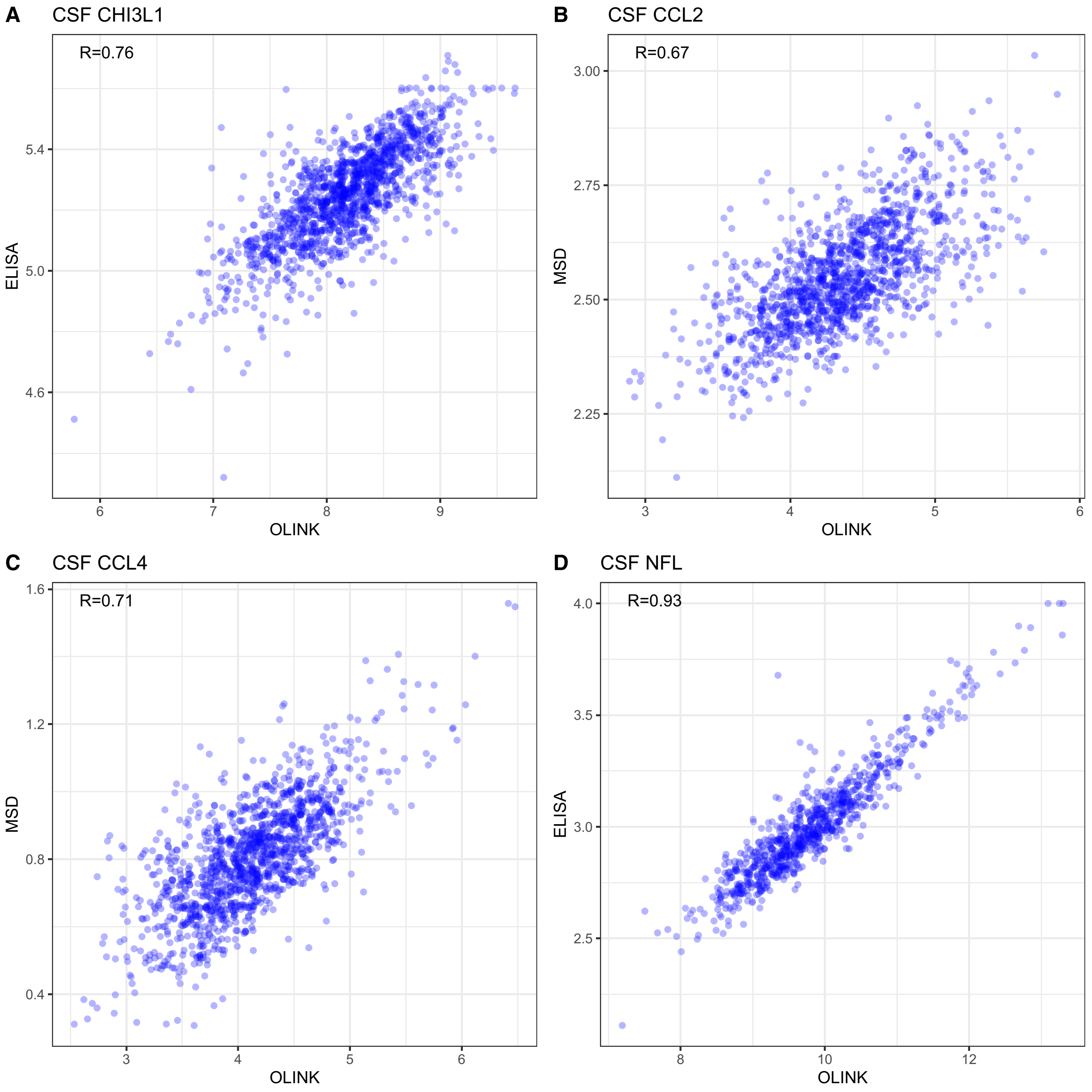


**Figure EV2. CSF biomarkers measured with orthogonal methods.**

A–D Between-assay correlations for CSF biomarkers measured with both OLINK methods (proximity extension assay) and orthogonal methods, for four proteins where this data was available (panel A: CHI3L1, panel B: CCL2, panel C: CCL4, panel D: NFL). pQTLs were identified for CHI3L1, CCL2 and CCL4, as described in the main manuscript. pQTLs identified by the orthogonal methods are included in Dataset EV2 (using alternative protein labels for the alternative assays: MCP1 for CCL2, MIP1b for CCL4, and YKL-40 for CHI3L1, rows marked yellow). For CCL2, the same genetic variant was identified (rs2228467, *trans*-pQTL) with both assays. For CCL4, one *trans*-pQTLs identified by proximity extension assay was validated (rs113341849) and one *cis*-pQTL (rs879571071) was identified, which was in LD with a *cis*-pQTL identified for the proximity extension assay (rs8064426, $R^2 = 0.200$, $D' = 0.687$). For CHI3L1, one *cis*-pQTL was also identified (rs4950928), which was in high LD with a *cis*-pQTL identified for the proximity extension assay (rs946262, $R^2 = 0.902$, $D' = 1.0$). For all these cases, the effect sizes of the pQTLs were similar, with stable direction of effects.

 © 2022 The Authors

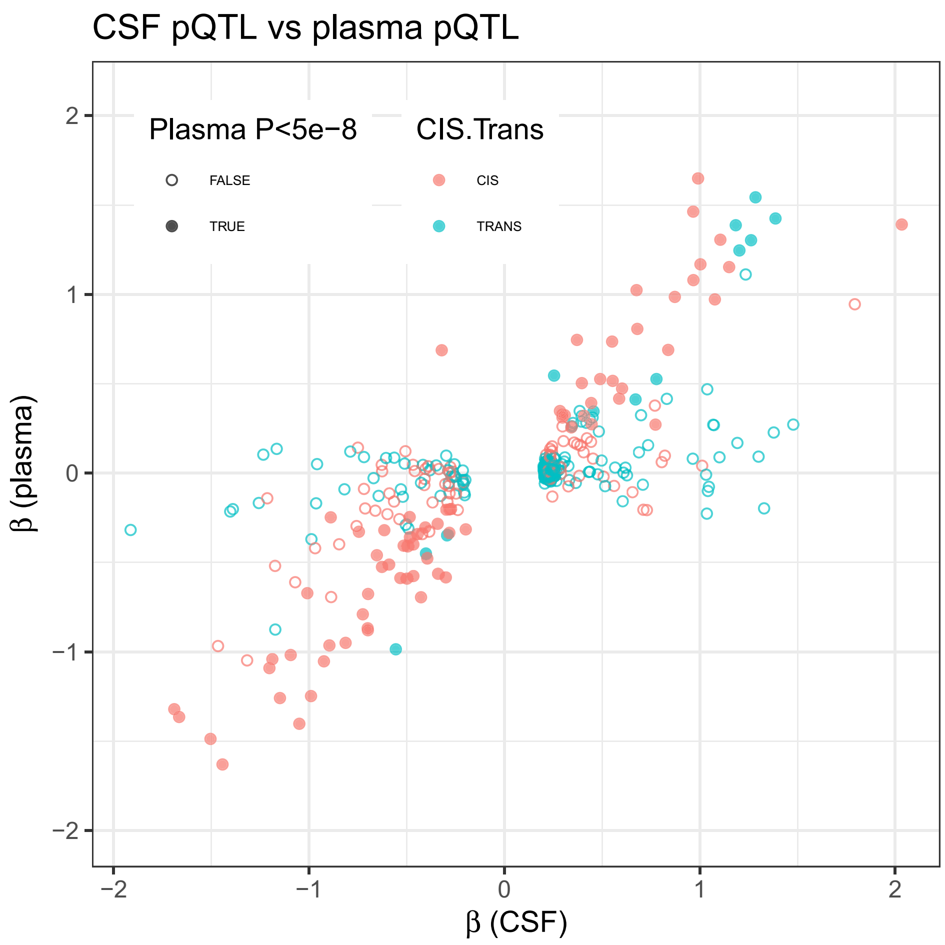


**Figure EV3. pQTLs in CSF and plasma.**

The figure shows the general relationship between pQTLs in CSF and plasma, for all pQTLs that were at least genome-wide significant ($P < 5e\text{-}8$) in CSF. The only pQTL which was significant after Bonferroni correction in CSF and genome-wide significant in plasma, where the direction of effect differed between the tissues, was a cis-pQTL for CXCL1, where the variant was associated with lower levels in CSF and higher in plasma.

© 2022 The Authors

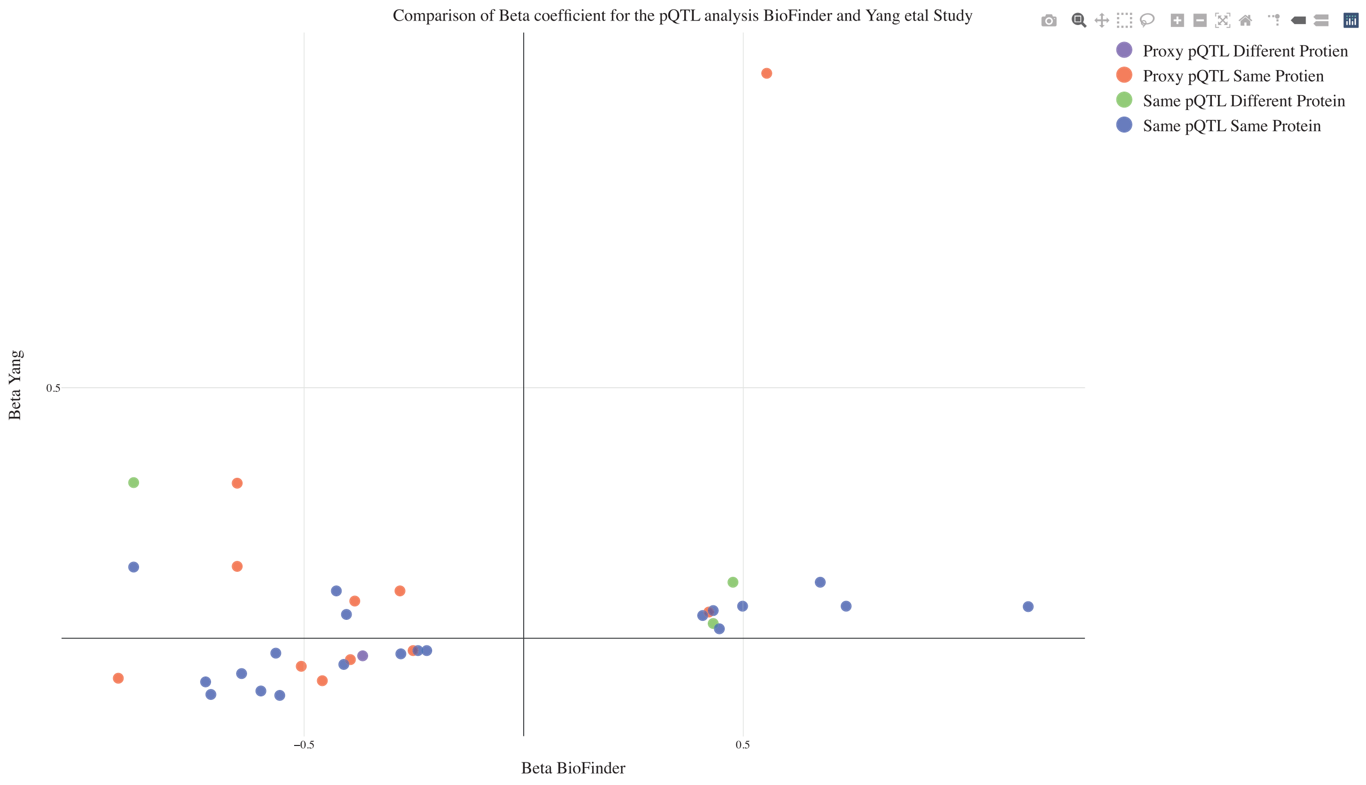


**Figure EV4. Replication of CSF pQTLs from Yang *et al* (2021).**

This figure shows a comparison between pQTLs that were identified both in the current study and in another recent publication on CSF pQTLs (Yang *et al*, 2021). Full interactive functionality is provided in the Source Data file "Fig EV4 Replication CSF pQTLs.html", which is available online.

Source data are available online for this figure.


© 2022 The Authors

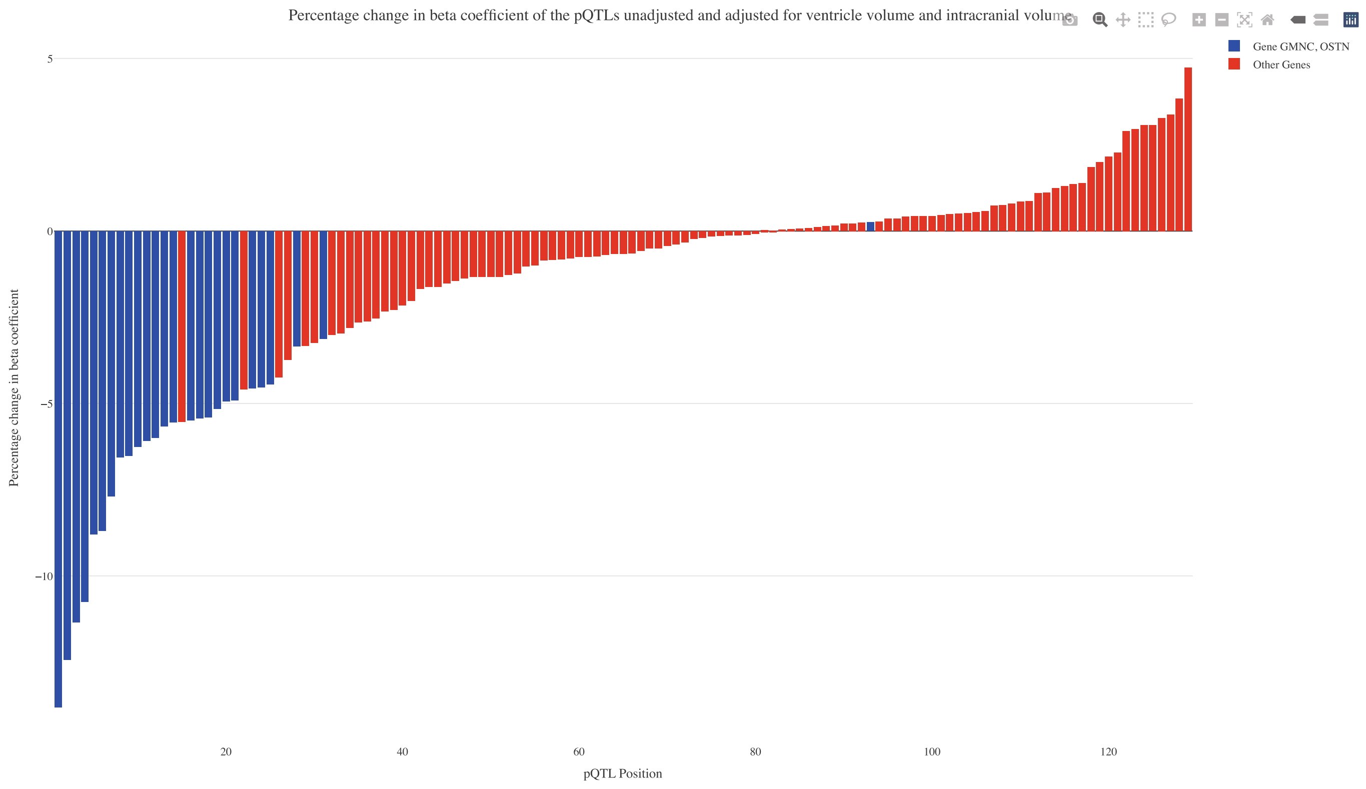


**Figure EV5. Effects on CSF pQTL from adjustment for ventricle volume.**

This figure shows the change in CSF pQTL β-coefficients with and without additional adjustment for ventricle volume and intracranial volume. pQTLs involving genetic variants in the *GMNC-OSTN* region on chromosome 3 are highlighted in blue. Note that these analyses were done on a subset of participants with MRI data. The plot (and corresponding Dataset EV14) is restricted to CSF protein-pQTL pairs that were at least genome-wide significant in the full cohort and at least significant with $P < 5 \times 10^{-6}$ in the MRI subcohort. Full interactive functionality is provided in the Source Data file “Fig EV5 Percent_Change_beta_ICV_VV.html”, which is available online.

Source data are available online for this figure.

© 2022 The Authors
