# Supplementary material for: The genetic regulation of protein expression in cerebrospinal fluid
Source: EMBO Mol Med. 2022 Dec 12;15(1):e16359. doi: 10.15252/emmm.202216359 (PMC9832827; doi:10.15252/emmm.202216359)
Supplement: Supplementary file 21 [file EMMM-15-e16359-s020.pdf]

EMBO Molecular Medicine

The genetic regulation of protein expression in cerebrospinal fluid

Oskar Hansson, Atul Kumar, Shorena Janelidze, Erik Stomrud, Philip Insel, Kaj Blennow, Henrik Zetterberg, Eric B. Fauman, Åsa Hedman, Michael Nagle, Christopher Whelan, Denis Baird, Anders Mälarstig, and Niklas Mattsson-Carlgren
DOI: 10.15252/emmm.202216359

Corresponding authors: Niklas Mattsson-Carlgren (niklas.mattsson-carlgren@med.lu.se) , Oskar Hansson (oskar.hansson@med.lu.se)

Review Timeline:

Submission Date:	23rd May 22
Editorial Decision:	12th Jul 22
Revision Received:	16th Sep 22
Editorial Decision:	8th Nov 22
Revision Received:	12th Nov 22
Accepted:	14th Nov 22

Editor: Jingyi Hou

Transaction Report:

(Note: With the exception of the correction of typographical or spelling errors that could be a source of ambiguity, letters and reports are not edited. Depending on transfer agreements, referee reports obtained elsewhere may or may not be included in this compilation. Referee reports are anonymous unless the Referee chooses to sign their reports.)

12th Jul 2022

Dear Dr. Mattsson-Carlgren,

Thank you again for submitting your work to EMBO Molecular Medicine. We have now heard back from three referees who agreed to evaluate your manuscript. As you will see from the reports below, the referees acknowledge the potential interest of the study. However, they raise a series of concerns, which we would ask you to address in a revision of the manuscript.

Without reiterating all the points raised in the reviews below, some of the more substantial issues are the following:

- Address Referee #1's major concerns #1 and #2.

- Considering the comments of Referee #2 regarding Yang et al, 2021, attention should be given to placing the findings in the context of existing literature and highlighting the novelty of the current study.

- Also, the discrepancy with Yang et al, 2021 should be better discussed.

Please feel free to contact me in case you would like to discuss in further detail any of the issues raised by the referees. We would welcome the submission of a revised version within three months for further consideration. Please note that EMBO Molecular Medicine strongly supports a single round of revision. As acceptance or rejection of the manuscript will depend on another round of review, your responses should be as complete as possible.

EMBO Molecular Medicine has a "scooping protection" policy, whereby similar findings that are published by others during review or revision are not a criterion for rejection. Should you decide to submit a revised version, I do ask that you get in touch after three months if you have not completed it to update us on the status.

We are aware that many laboratories cannot function at full efficiency during the current COVID-19/SARS-CoV-2 pandemic and have therefore extended our "scooping protection policy" to cover the period required for a full revision to address the experimental issues. Please let me know should you need additional time, and also if you see a paper with related content published elsewhere.

Please read below for important editorial formatting and consult our author's guidelines for proper formatting of your revised article for EMBO Molecular Medicine.

Use this link to login to the manuscript system and submit your revision: https://embomolmed.msubmit.net/cgi-bin/main.plex

I look forward to receiving your revised manuscript.

Kind regards,
Jingyi

Jingyi Hou
Editor
EMBO Molecular Medicine

***** Reviewer's comments *****

Referee #1 (Comments on Novelty/Model System for Author):

Summary
The main strength of this work is that it utilized CSF samples for protein detection, which better reflects the brain pathology compared to plasma and is easy to obtain. And the study is well designed with standard pipeline.

Referee #1 (Remarks for Author):

Suggestions for improvement
Major

1. Did the authors adjust for disease status in genome-wide association analysis to identify pQTLs? Please specify. If disease status were not adjusted, the authors should consider the effects those diseases have on protein level. Since the cohort included patients diagnosed with nervous system diseases, it might confound the results. Also, sensitivity analyses on cognitively healthy participants would be useful.

2. The author mainly utilized MR to investigate the effect of CSF protein on disease traits. MR is a good method for analysis, but it has some limitations. For example, the instrumental variable may be outlier and missense variants may result in artificial pQTL, in which case MR might not be able to obtain an accurate estimate. Since the cohort includes people with disease diagnosis, the author can consider performing clinical analyses of the association between protein levels and disease prevalence. If the results of clinical analysis and MR analysis are consistent, the conclusion could be more solid and convincing.

3. Many of the shared pQTL between this work and Yang et al's presented inconsistent directions (Table S3). Inconsistent directions are common in QTL studies. As described by the authors, aptmer- and antibody-based technologies provide different information, which is a possible reason. However, the phenomenon that directions differ among studies might hugely influence the reliability of subsequent analysis on the correlation with diseases (e.g., MR). I hope the authors could provide discussion about that which technology would be more reliable for pQTL study and that how to improve consistency in future researches.

Minor
1. How did the authors find 176 independent genetic loci and what's the r2 of independent genetic loci? I did not find detailed description. And did the authors perform conditional analyses to find multiple conditionally significant associations? (https://doi.org/10.1038/s41586-018-0175-2)

2. The authors performed many MR analyses with rs429358 as instrumental variable (Table S8-9). However rs429358 is correlated with multiple diseases (such as AD) and risk factors and affects many protein levels (Table S2), which is a loci with high pleiotropy and thus not appropriate as an instrumental variable in MR.

Referee #2 (Remarks for Author):

This is a study looking a genetic determinants of CSF proteins. The sample size is quite large but the number of measure proteins is relative small compared with previous studies. The authors find 139 pQTLs for 148 proteins that used for comparison with eQTL and for MR. In general, the analyses (see specific comments below) are well performed, but the results or the workflow are in general not novel and larger studies have already been performed, so it is not clear what this manuscript adds. The association of pQTLs in CH13L1, GRN, APOE have already reported. The overall manuscript follows the manuscript from Yang et al, with similar sections, and similar findings.

Specific comments:
One of the major weaknesses of this study is the lack of replication in independent datasets or using al orthogonal approach. In addition, this dataset is enriched for individuals with neurodegenerative diseases and it is not clear if any of the pQTLs are disease-specific.
Many findings are already reported. The authors used the GWAS catalog on May 2021 to compare new results. This is a very active field a very large plasma pQTL have published recently. Although it may not be fair to ask the authors check manuscript that were published when this manuscript was ready to be submitted, May 2021 is more than one year old, and the author should check more recently.

The analyses of the pQTL vs eQTL is interesting, but the analyses do not go far enough. It is not clear reading the text the overlap between p and eQTL. There is not proper colocalization analyses, and the it is not a clear explanation why almost 1/3 of the QTLs are in opposite direction. The GTEX includes small brain dataset, and it is not clear why the authors did not use the meta-brain data for these analyses.

The association of GMNC with ventricle volume does not have strong rationale. The authors do not say what proteins have trans pQTL for this region, which makes more complicated to interpret this section. The association of this variant is barely associated with ventricle volume (p=0.007), which will not pass any multiple test correction.

The Mendelian randomization analyses do not cover potential pleiotropy , even the authors only focused on cis signals. The APOE region is a known pleiotropic region, same as several others. The authors should clarify how the address this issue and how many IV were used on each of these analyses. In addition many of these findings were already reported by Yang et al. the authors should make clear what has been already reporter and what not
It is not clear if FDR correction for pQTL is adequate as this is done within a protein and does not take into account the multiple proteins measure

Referee #3 (Remarks for Author):

In this manuscript the authors describe the identification of genetic variants that associate with levels of protein detected in CSF and plasma in a large cohort of ~1500 individuals and further investigate the potential confounding role of ventricular volume. Using Mendelian Randomization causal roles for certain proteins were predicted for several neurological conditions. Such work is important to better understand both protein biomarkers of disease and the contributions of genetics to such biomarkers that can improve diagnosis, detect disease pre-clinically, and to potentially identify new therapeutic targets. There are several aspects of the manuscript that could use clarification to make the design and conclusions more valuable to the readers and community. My suggestions for improvement are below:

Introduction:
1 - The introduction is cursory and does not comprehensively introduce what is currently known (only cites two existing pQTL studies). A more thorough review of the state of field and the benefits of pQTL analysis would be preferred (rather than a restating of what is already in the abstract and detailed in the methods)
Methods:
1 - Is gene distance the best method for determining gene linked to a variant? Given the complexity of gene regulation at the expression and post-transcriptional landscape this annotation method does not take into account long range enhancers for example or other noncoding regulatory elements. The pQTLs should be annotated to include any known interactions (Hi-C for example) or known enhancers for more accurate annotation.
Results:
1 - A table/supplementary table of the subject demographics would be helpful. Including for example the ages, sexes, of the various neurological conditions (as written there is a just a list but no details).

2 - The distinction between Bonferroni pQTL and genome-wide pQTL is not well established and no interpretation is provided. Should only Bonferroni be presented and believed/trusted? Why include both in the results?
3 - Only 15 pQTL overlap with previous study. This seems incredibly lower than expected.
4 - In general, the result section is scattered and lacking overall focus. Makes it difficult to determine the theme throughout and at times reads as a laundry list of analyses and statistical test. A reorganization of this section with a common theme
Discussion:
1 - not enough attention is paid to the discrepancy with existing pQTL studies. Why so few overlaps? Is that overlap expected simply by chance?
2 - the significance of such a study is not made clear in the discussion. How does this lead to therapeutics? Are any new targets identified? Given that well known targets (e.g. APOE) are identified, does this add anything significant?

Referee #1 (Comments on Novelty/Model System for Author):

Summary
The main strength of this work is that it utilized CSF samples for protein detection, which better reflects the brain pathology compared to plasma and is easy to obtain. And the study is well designed with standard pipeline.

Answer: We appreciate this kind comment about our work.

Referee #1 (Remarks for Author):

Suggestions for improvement
Major
1. Did the authors adjust for disease status in genome-wide association analysis to identify pQTLs? Please specify. If disease status were not adjusted, the authors should consider the effects those diseases have on protein level. Since the cohort included patients diagnosed with nervous system diseases, it might confound the results. Also, sensitivity analyses on cognitively healthy participants would be useful.

Answer: We adjusted for disease status in the original genome-wide association analyses. We have now revised the main text to more clearly describe the covariates included in our analyses. We have also conducted sensitivity analyses in cognitively healthy participants only. The results from these sensitivity analyses, which are included in the paper, correlated strongly with the main results. We have added this text to the results (page 13):

"The genome-wide analyses were adjusted for diagnostic group, but in a sensitivity analysis we repeated the CSF analyses in the subgroup of cognitively normal controls, with similar results as the main analysis (Appendix Figure S2)."

This is Appendix Figure S2 (original results on y-axis, results in the controls only on x-axis):

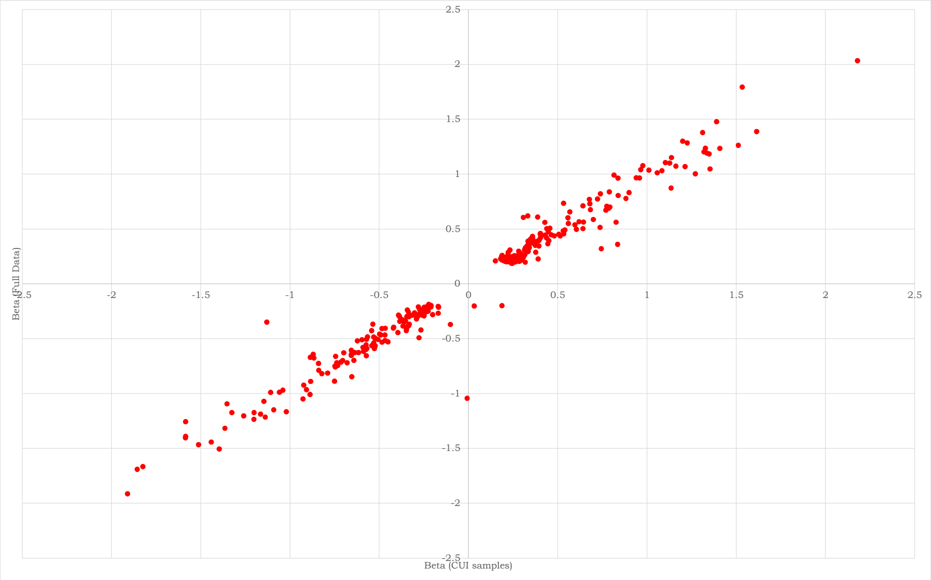

2. The author mainly utilized MR to investigate the effect of CSF protein on disease traits. MR is a good method for analysis, but it has some limitations. For example, the instrumental variable may be outlier and missense variants may result in artificial pQTL, in which case MR might not be able to obtain an accurate estimate. Since the cohort includes people with disease diagnosis, the author can consider performing clinical analyses of the association between protein levels and disease prevalence. If the results of clinical analysis and MR analysis are consistent, the conclusion could be more solid and convincing.

Answer: We agree that pQTLs that tag missense variants can sometimes reflect epitope effects rather than true effects on protein levels. Epitope effects may indeed confound Mendelian randomization analyses but would largely drive the MR result towards a null result rather than inflating the risk of a true positive., To increase the risk of weak epitope effects we only selected variants that were significant after Bonferroni correction as instruments in MR. To address the concern around epitope effects, we now include annotation of predicted functional consequence for the pQTLs used as instrumental variables in the MR table (Table EV12). Three of the variants were missense (APOE, SIGLEC9, FLRT2), while the others were intronic/UTR-3/intergenic. We now include the following text in the limitations section (page 26):

"We cannot exclude that the MR analysis may be affected by "pseudo pQTLs" or pQTLs driven by epitope effects, i.e., that the assay detects protein with missense variant differently (especially for APOE, SIGLEC9, FLRT2, which were missense variants)."

We also appreciate the suggestion to investigate whether a protein found to be causal using MR also show "observational" associations with disease. In our cohort, we could evaluate if CSF APOE, CD33 and GRN (implicated by MR for AD) differed between AD and controls; if CSF MMP10 (implicated by MR for preclinical AD) differed between preclinical AD and other cognitively normal subjects; and if CSF CD38, GPNMB and ADAM15 (implicated by MR for PD) differed between PD and controls. We found that CSF GRN levels differed significantly between AD and controls; CSF MMP10 differed significantly between preclinical AD and other cognitively normal subjects; and CSF CD38 and GPNMB differed significantly between PD and controls. In our opinion, this supports the MR findings for these proteins. These results are now described in the main text, and included as a new Table EV13. Hypothetically, it is possible that the lack of significant associations for CD33 with AD and ADAM15 with PD could be disease-stage dependent, since causal biomarkers may not always show cross-sectional differences during the later stages of the disease. We include this text in the main manuscript, results section (page 18):

"For the AD, preclinical AD, and PD findings, we had the opportunity to test for observational differences between diagnostic groups in CSF biomarker levels for the proteins implicated in the MR analysis (AD: APOE, CD33 and GRN; Preclinical AD: MMP-10; PD: CD38, GPNMB, ADAM15). We found significant effects for GRN in AD, for MMP-10 in preclinical AD, and for CD38 and GPNMB in PD, supporting the MR findings (Table EV13)."

We include this text in the discussion section (page 22-23):
"Several CSF proteins identified in the MR analysis for AD (GRN), preclinical AD (MMP-10), and PD (CD38 and GMBP) were also validated with observational differences between patients and controls. We did not find altered levels for CD33 or APOE (for AD) or ADAM15

(for PD). Hypothetically, this may be disease-stage dependent, since causal biomarkers may not always show cross-sectional differences during the later stages of the disease."

We include this line in the abstract (page 3):
"CSF GRN, MMP-10 and GPNMB were altered in Alzheimer's disease, preclinical Alzheimer's disease and Parkinson's disease respectively, supporting their role in these conditions."

3. Many of the shared pQTL between this work and Yang et al's presented inconsistent directions (Table S3). Inconsistent directions are common in QTL studies. As described by the authors, aptmer- and antibody-based technologies provide different information, which is a possible reason. However, the phenomenon that directions differ among studies might hugely influence the reliability of subsequent analysis on the correlation with diseases (e.g., MR). I hope the authors could provide discussion about that which technology would be more reliable for pQTL study and that how to improve consistency in future researches.

Answer: We agree that it is interesting to further discuss the differences between these two studies and the technologies used. We note that a recent large study by Katz et al (Science Advances 2022; 8: 33) directly compared SomaScan with OLINK and found considerable differences in proteomic studies done with these two technologies. Their evidence supported more reliable protein target specificity and a higher number of phenotypic associations for the OLINK platform. We now discuss these findings in the revised version of the paper. We include this new text in the introduction (page 4):

"The largest previous study on CSF pQTLs used an aptamer-based approach (Yang et al, 2021). Recent comparisons between aptamer- and antibody-based pQTL studies highlight that the technology used for protein quantification could have a significant impact on the findings, with antibody-based methods being more specific (Katz et al, 2022)."

We include this text in the discussion (page 25):
"A recent large-scale study directly compared the aptamer-based SomaScan technology with antibody-based PEA and found considerable differences in results (Katz et al, 2022), which is in accordance with our findings. Importantly, Katz et al found evidence supporting more reliable protein target specificity and a higher number of phenotypic associations for the PEA assays, used in the current study."

Minor
1. How did the authors find 176 independent genetic loci and what's the r2 of independent genetic loci? I did not find detailed description. And did the authors perform conditional analyses to find multiple conditionally significant associations? (https://doi.org/10.1038/s41586-018-0175-2)

Answer: We have now clarified this in the Methods (page 9) :
"Linkage disequilibrium (LD) analysis was performed to select the lead SNP within the LD region, using a correlation of R2 = 0.1 to identify independent genetic loci."

With regards to conditional analyses for multiple conditionally significant associations, the statistical power for many rounds of condition with our sample size would be low.

2. The authors performed many MR analyses with rs429358 as instrumental variable (Table

S8-9). However rs429358 is correlated with multiple diseases (such as AD) and risk factors and affects many protein levels (Table S2), which is a loci with high pleiotropy and thus not appropriate as an instrumental variable in MR.

Answer: We appreciate the reviewer comment. Our primary MR analysis focused on cis-pQTLs (described extensively in the main text, and in Table 1, and EV Tables), where rs429358 is valid as an instrumental variable. In the original version of the paper, we also conducted MR analysis for the trans-pQTLs. We agree with the reviewer that such a trans-pQTL MR analysis for rs429358 carries a risk of introducing horizontal pleiotropy. We have therefore removed all trans-pQTL MR analysis from the paper.

Referee #2 (Remarks for Author):

This is a study looking a genetic determinants of CSF proteins. The sample size is quite large but the number of measure proteins is relative small compared with previous studies. The authors find 139 pQTLs for 148 proteins that used for comparison with eQTL and for MR. In general, the analyses (see specific comments below) are well performed, but the results or the workflow are in general not novel and larger studies have already been performed, so it is not clear what this manuscript adds. The association of pQTLs in CH13L1, GRN, APOE have already reported. The overall manuscript follows the manuscript from Yang et al, with similar sections, and similar findings.

Answer: We appreciate the comments. In the revised version of the paper, we now highlight that Yang et al used the SomaScan aptamer-based assays, while we used OLINK antibody-based assays, which is an important novelty. As recently demonstrated (Katz et al, 2022), SomaScan has comparably lower specificity than the OLINK assays. Another recent study from Kári Stefánsson and the deCODE group compared plasma pQTLs detected via OLINK and SomaScan. That study showed that OLINK showed better suitability for pQTL detection (https://www.biorxiv.org/content/10.1101/2022.02.18.481034v1).
There is therefore a need for CSF pQTL studies with assays with greater specificity, such as the OLINK assays. Our study also includes other novel experiments compared to the study by Yang et al, including a section on exploring ventricle volume measured by brain MRI as a confounder for CSF pQTLs. We describe these issues throughout the introduction and discussions sections.

Specific comments:
One of the major weaknesses of this study is the lack of replication in independent datasets or using al orthogonal approach. In addition, this dataset is enriched for individuals with neurodegenerative diseases and it is not clear if any of the pQTLs are disease-specific.
Many findings are already reported. The authors used the GWAS catalog on May 2021 to compare new results. This is a very active field a very large plasma pQTL have published recently. Although it may not be fair to ask the authors check manuscript that were published when this manuscript was ready to be submitted, May 2021 is more than one year old, and the author should check more recently.

Answer: The pQTL analyses were adjusted for diagnosis in the original version of the paper. We have now explained this in the Methods section (page 9). We now also provide a sensitivity analysis restricted to the control population, which showed very similar results as the main analysis (page 13). Please see the response to reviewer #1, comment #1.

We agree that there are other plasma pQTL studies, but there is a scarcity of CSF pQTL studies, especially with highly specific assays for a large number of proteins, as performed here. We have added this section to the introduction, and updated the text with additional citations (page 4):

"While studies of the genetic control of the CSF proteome may yield insights into brain disease mechanisms and potential treatments, only a few such studies have been performed to date on larger sets of proteins (Sasayama et al, 2019; Yang et al, 2021; Kunkle et al, 2019), or focused on a few specific disease-associated proteins (Deming et al, 2017; Maxwell et al, 2018). In contrast, several studies in blood plasma have consistently shown that genetic variants associated with protein levels, known as protein quantitative trait loci (pQTL), using

both aptamer-based (Sun et al, 2018; Ferkingstad et al, 2021) and antibody-based assays (Folkersen et al, 2020) are common and can explain up to 30 % of the protein variance. Identification of pQTLs makes it possible to test if proteins are likely to be causal for development of human diseases (hence nominating them as candidate drug targets). For neurological diseases, more pQTL studies are needed on proteins in CSF rather than plasma to identify disease pathways and potential drug targets. The largest previous study on CSF pQTLs used an aptamer-based approach (Yang et al, 2021). Recent comparisons between aptamer- and antibody-based pQTL studies highlight that the technology used for protein quantification could have a significant impact on the findings, with antibody-based methods being more specific (Katz et al, 2022)."

We have updated all EBI GWAS queries. This updated work was done by Eric Fauman, who is now added as a co-author on the paper. In summary, the updated GWAS query demonstrates that this study adds several novel CSF pQTLs, as described below. All results for the updated EBI-GWAS query are included in detail in Tables EV4-7. We have added this text to the Methods section (page 10):

"GWAS catalogue comparisons
In order to assess the relevance and novelty of our pQTLs we compared all our lead SNPs to all the lead SNPs reported in the GWAS catalogue. We downloaded all the lead SNPs from the GWAS catalogue (https://www.ebi.ac.uk/gwas/api/search/downloads/alternative). We retained all entries that were within 250 Kbp of any of our lead SNPs. For each of our lead SNPs we recorded 1) the total number of GWAS catalogue entries, 2) the total number of pQTLs, 3) the total number of distinct protein biomarkers, 4) the total number of "CNS disease" traits and 5) the total number of distinct "CNS disease" traits within 250 Kbp of that lead SNP. A GWAS association was defined as representing a "CNS disease" trait if one or more of the Experimental Factor Ontology terms assigned by the GWAS catalogue curators was a descendant of the term "central nervous system disease" in that ontology (http://www.ebi.ac.uk/efo/EFO_0009386). There are 2717 descendants of "CNS disease" although certainly they are not all represented in the GWAS catalogue.
GWAS associations in the GWAS catalogue were defined as representing pQTLs through a laborious manual review of all 300 000+ GWAS catalogue associations. An association was defined as a pQTL if the trait represents the abundance of the protein product of a human gene. Each association defined as a pQTL was tagged with the human gene or genes encoding the protein (or subunits of the protein if it is a heteromultimer, such as hemoglobin). While most published pQTLs represents protein abundance from plasma or serum samples, a small number represent prior CSF pQTL studies. These were identified by the presence of the word "CEREBROSPINAL" in the "DISEASE/TRAIT" field in the GWAS catalogue. Novelty of the CSF pQTLs identified in the current study was assessed on multiple levels, as described in the Results."

We have added this text to the Results section (page 15):
"We queried the EBI-GWAS catalogue (September 2022) for all Bonferroni significant CSF pQTLs identified in this study, as described in the Methods section. Novelty of the CSF pQTLs was assessed on multiple levels, depending on whether we found in the GWAS catalogue any prior pQTL in the locus, a prior pQTL for the protein we observed at the locus, any prior pQTL in CSF at the locus, or a prior pQTL in CSF for the protein we observed at the locus. The result of this novelty assessment is provided in Table EV4. A full summary of the GWAS catalogue hits is provided in Table EV5. Detailed results for CNS disease hits and pQTL hits are provided in Table EV6 and Table EV7, respectively. In summary, among all CSF pQTL-

protein pairs included in this analysis, only 19 (10.9%) were validated from previous publications (the pQTL was reported before for the same protein in CSF), 82 (47.1%) were novel in CSF but a pQTL in the same region had been reported before for the same protein in serum or plasma, 43 (24.7%) were novel for the identified protein, but the locus had been associated before with other proteins in CSF, 27 (15.5%) were novel for the identified protein, but the locus had been associated before with other proteins in serum or plasma, and 3 (1.7%) were completely novel (no pQTL reported before for the locus for any protein in any tissue)."

The analyses of the pQTL vs eQTL is interesting, but the analyses do not go far enough. It is not clear reading the text the overlap between p and eQTL. There is not proper colocalization analyses, and the it is not a clear explanation why almost 1/3 of the QTLs are in opposite direction. The GTEX includes small brain dataset, and it is not clear why the authors did not use the meta-brain data for these analyses.

Answer: We agree that we did not extensively describe the pQTL vs eQTL results in the main text. We provide complete results for these analyses in the supplementary tables EV5 and EV6. We want to highlight that we have used both the meta-brain data (Sieberts et al, 2020), shown in Table EV9 (and Figure 5), as well as GTEx data, shown in Table EV10 (and Appendix Figure S4). We also point out in the text that these sources provided partly different information (page 16-17). We have now added a co-localization analysis for CSF cis-pQTLs vs eQTL using the Sieberts et al meta-analysis data. We have added these results as new columns in Table EV6. We noted that QTLs with discordant direction tended to have lower co-localization probability compared to QTLs with concordant directions (among those with discordant signs, only 4/18 [22%] had high (at least 0.85) co-localization probability, compared to 18/35 [51 %] among those with concordant signs). One possibility is that CSF pQTLs with low co-localization probability with eQTLs regulate protein levels through mechanisms distinct from direct effects on transcription. Regarding mismatch between the QTLs, we also note that eQTL and disease-associated loci may have fundamental differences, as recently discussed (Mostafavi et al, https://www.biorxiv.org/content/10.1101/2022.05.07.491045v1). In the revised version of the paper, we also highlight and cite a recent plasma pQTL study, which also showed the occurrence of discordant directions of effect for molecular QTLs on gene expression and protein levels, and discussed possible reasons for such results (Sun et al, 2022).

We have added this text in the Results part of the manuscript (page 16):
"Co-localization experiments showed that concordant QTLs tended to have higher co-localization probability (18/35 had co-localization probability of at least 0.85), while discordant QTLs tended to have lower co-localization probability (only 4/18 had co-localization probability of at least 0.85)."

We have added this section to the discussion of the manuscript (page 26):

"In a comparison between CSF pQTLs and brain eQTLs, we noted that there were both concordant associations, supporting direct links between gene transcription and translation, as well as discordant associations. Discordant associations between pQTLs and eQTLs have reported to varying degrees before (Sun et al, 2022), and may hypothetically reflect biological pathways with negative feedback loops that regulate protein levels. As recently pointed out, eQTL and disease-associated loci may have fundamental differences, contributing to different results with these approaches(Mostafavi et al, 2022)."

The association of GMNC with ventricle volume does not have strong rationale. The authors do not say what proteins have trans pQTL for this region, which makes more complicated to interpret this section. The association of this variant is barely associated with ventricle volume (p=0.007), which will not pass any multiple test correction.

Answer: The proteins with trans-pQTLs in the GMNC-OSTN region are listed in Table EV3 together with all pQTL findings, but for convenience we now also provide a new separate Table EV14 restricted to only the 36 trans-pQTLs with associations to GMNC/OSTN, with full details, including protein names. We have now also updated the EBI-GWAS query (September 2022) for published associations linked to GMNC/OSTN. The updated results (available in Table EV15) continue to support that this genomic region is linked to brain morphology. Out of 29 unique traits identified, 20 were brain-related and most of those were morphology related (mainly volumes or thicknesses of different brain regions and ventricles, along with white matter microstructure traits). The paper (Results section) now includes this text (page 20):

"We queried the EBI GWAS catalogue (September 2022) for previous associations with variants mapped to GMNC or OSTN. There were 77 hits in total (Table EV15), including 42 unique genetic variants. Twenty out of 29 unique reported traits were brain-related, mainly comprising volumetric brain measures, including of ventricle volume or white matter lesions, supporting the relevance of this genomic region for CNS-related molecular pathways. Of the eight GMNC/OSTN-related variants that we identified, there were three cases (rs201663722, rs4308324, and rs71635338) with exact matches to variants previously described to be associated with brain volumetric measures."

With regards to the association between rs71635338 and ventricle volume, this was a directed statistical test for this particular variant (the most common trans-pQTL SNP in the study), driven by the specific hypothesis that brain ventricle volume would be associated with variants in this region, as informed by previous genetic associations at genome-wide levels of statistical significance, for example Zhaeo et al, Nature Genetics 2019 and Vojinovic et al, Nature Communications 2018. Multiple test correction was therefore not applicable. We include citations in the text, which now reads (Results section) (page 20):

"Given the importance of the GMNC/OSTN region for CSF trans-pQTLs and previous links between genetic variants in this region and brain ventricle volume (Vojinovic et al, 2018; Zhao et al, 2019), we hypothesized that brain ventricle volume may be a confounder for CSF pQTLs (due to dilution effects on proteins)."

We also clarify in the same section, regarding the association between rs71635338 and ventricle volume (page 21):
"Note that this was a single test, based on an a-priori hypothesis, whereby correction for multiple comparisons was not applicable."

Furthermore, we note that a very recent study in Acta Neuropathologica (Jansen et al, "Genome-wide meta-analysis for Alzheimer's disease cerebrospinal fluid biomarkers", 2022, online head of print, doi: 10.1007/s00401-022-02454-z) found that genetic variants in the GMNC-OSTN region that were associated with smaller ventricles were also associated with increased CSF P-tau levels. Jansen et al wrote that "The formerly reported directions of

effect of GMNC and C16orf95 for ventricular volume are counterintuitive. For both loci the allele that associated with an increase in pTau pathology in our dementia cohorts associated with a smaller ventricular volume, implying less neurodegeneration." We suggest that our study resolves this supposedly counterintuitive finding. Our results, across several CSF proteins, suggest that the effects on ventricular volume from genetic variants in the GMNC-OSTN region confound the associations between the genetic variants and CSF biomarkers. Specifically, the associations between CSF biomarker levels and these genetic variants are likely influenced by CSF dilution and concentration factors (a genetic variant which results in smaller ventricles leads to reduced CSF volume and thereby higher CSF protein levels through increased concentration). Taken together, we believe that these findings make it important to highlight that ventricular volume may confound associations between some genetic variants and CSF biomarker levels. This is an important novelty of this paper. We have added the following text in the discussion section of the paper: (page 24)

"Recently, it was reported that genetic variants in the GMNC-OSTN region on chromosome 3 (described in detail in this study) were associated both with smaller ventricles and with increased CSF levels of the AD biomarker P-tau (Jansen et al, 2022). This finding may first be perceived as counterintuitive, since smaller ventricles is generally thought to indicate less neurodegeneration, and increased CSF P-tau is generally associated with more neurodegeneration. However, the findings in our current study suggest that the association may have been confounded by the genetic effects on brain anatomy, causing differences in dilution and concentration across a range of CSF biomarkers. We noted that variants in the GMNC-OSTN region were common among the CSF pQTLs where ventricular volume had large confounding effects (Figure EV5). Future studies of the genetic regulation of the CSF proteome may therefore benefit from an integration of brain imaging, as demonstrated here, to adjust for such confounding effects."

Comment: The Mendelian randomization analyses do not cover potential pleiotropy , even the authors only focused on cis signals. The APOE region is a known pleiotropic region, same as several others. The authors should clarify how the address this issue and how many IV were used on each of these analyses. In addition many of these findings were already reported by Yang et al. the authors should make clear what has been already reporter and what not
It is not clear if FDR correction for pQTL is adequate as this is done within a protein and does not take into account the multiple proteins measure

Answer: We have now removed all trans-pQTL MR analyses from the paper, which eliminates the problem of pleiotropy for the APOE region. For the cis-pQTL MR analyses, only one SNP was included per exposure (wherefore the Wald ratio test was used). We followed up with colocalization analyses to guard against potential pleoiotropy. With regards to the correction for multiple comparisons, it was done over the entire set of MR analyses. MR analysis is not sensitive to vertical pleiotropy, which we see for the ApoE cis-pQTL but also for other cis-pQTLs in our study.

In the previous version of the paper, we included a comprehensive comparison of our pQTL findings with the Yang et al findings. For outcomes that were common between our CSF studies (AD, PD and ALS), we now also compare our MR results directly with the Yang et al findings. We note that (on gene-protein level) the finding for CD33 and AD overlap between the studies. The other findings in Yang et al and our study are unique to the studies. This may be partly because we study complementary outcomes, and partly because we use different assays with SomaScan in Yang et al and OLINK in our study, as discussed in response to

other comments above. There is also a possibility that differences between our study and Yang et al could partly be cohort dependent. We include this text in the revised version (Results section, page 19):

"We directly compared our MR findings with the findings in Yang et al (Yang et al, 2021) which included MR findings for CSF pQTLs and the outcomes AD, PD, ALS, stroke and FTD. Among our gene-level findings, the causal association between CD33 (also known as Siglec-3) and AD was replicated between the studies. The other MR findings were unique to our study and to those reported by Yang et al, respectively."

In the discussion section, we have added this line (page 23):
"We note that another recent CSF pQTL study also implicated CD33 for AD by MR analysis(Yang et al, 2021), while several other proteins were uniquely implicated in this study versus in Yang et al.."

Referee #3 (Remarks for Author):

Comment: In this manuscript the authors describe the identification of genetic variants that associate with levels of protein detected in CSF and plasma in a large cohort of ~1500 individuals and further investigate the potential confounding role of ventricular volume. Using Mendelian Randomization causal roles for certain proteins were predicted for several neurological conditions. Such work is important to better understand both protein biomarkers of disease and the contributions of genetics to such biomarkers that can improve diagnosis, detect disease pre-clinically, and to potentially identify new therapeutic targets. There are several aspects of the manuscript that could use clarification to make the design and conclusions more valuable to the readers and community. My suggestions for improvement are below:

Comment: Introduction:
1 - The introduction is cursory and does not comprehensively introduce what is currently known (only cites two existing pQTL studies). A more thorough review of the state of field and the benefits of pQTL analysis would be preferred (rather than a restating of what is already in the abstract and detailed in the methods)

Answer: We have revised the introduction, highlighting additional pQTL studies and the benefits of pQTL analyses. The introduction now includes this text (page 4):

"While studies of the genetic control of the CSF proteome may yield insights into brain disease mechanisms and potential treatments, only a few such studies have been performed to date on larger sets of proteins (Sasayama et al, 2019; Yang et al, 2021; Kunkle et al, 2019), or focused on a few specific disease-associated proteins (Deming et al, 2017; Maxwell et al, 2018). In contrast, several studies in blood plasma have consistently shown that genetic variants associated with protein levels, known as protein quantitative trait loci (pQTL), using both aptamer-based (Sun et al, 2018; Ferkingstad et al, 2021) and antibody-based assays (Folkersen et al, 2020) are common and can explain up to 30 % of the protein variance. Identification of pQTLs makes it possible to test if proteins are likely to be causal for development of human diseases (hence nominating them as candidate drug targets). For neurological diseases, more pQTL studies are needed on proteins in CSF rather than plasma to identify disease pathways and potential drug targets. The largest previous study on CSF pQTLs used an aptamer-based approach (Yang et al, 2021). Recent comparisons between aptamer- and antibody-based pQTL studies highlight that the technology used for protein quantification could have a significant impact on the findings, with antibody-based methods being more specific (Katz et al, 2022)."

Comment: Methods:
1 - Is gene distance the best method for determining gene linked to a variant? Given the complexity of gene regulation at the expression and post-transcriptional landscape this annotation method does not take into account long range enhancers for example or other noncoding regulatory elements. The pQTLs should be annotated to include any known interactions (Hi-C for example) or known enhancers for more accurate annotation.

Answer:
We now refer to the recent paper by Fauman & Hyde 2022 (cited in the Methods) on gene distance for annotations. However, we have also performed a chromatin-based annotation. In summary, among all significant (at $p < 1.25e-10$) pQTLs- protein pairs we performed

chromatin-based annotation for 142 unique pQTLs, to identify the relevant gene. The genes identified via distance-based and chromatin-based methods were similar for 120 unique pQTLs. There was a mismatch of genes for 3 pQTLs identified via both methods. For 19 pQTLs, there were no genes reported via the chromatin-based method. Detailed chromatin-based annotation and promoter region information is provided in (Table EV3) .

We added this text to the results (page 14):
"Our main method to annotate pQTL genes was by genomic distance, but annotation through chromatin interaction analysis yielded largely overlapping results (Table EV3)."

Comment: Results:
1 - A table/supplementary table of the subject demographics would be helpful. Including for example the ages, sexes, of the various neurological conditions (as written there is a just a list but no details).

Answer: We have added a demographics table structured by diagnosis (Table EV2).

2 - The distinction between Bonferroni pQTL and genome-wide pQTL is not well established and no interpretation is provided. Should only Bonferroni be presented and believed/trusted? Why include both in the results?

Answer: We appreciate this comment. We have now removed all main text about the genome-wide pQTL findings, to focus the results on the findings that were significant after Bonferroni correction. We let the genome-wide results remain available in Table EV3, to facilitate comparisons with other studies as well as future meta-analyses.

3 - Only 15 pQTL overlap with previous study. This seems incredibly lower than expected.

Answer: Please see responses to previous comments for discussion about the differences between this study and Yang et al. One important point is that the two studies used different assays with SomaScan in Yang et al and OLINK in our study. A recent large-scale study by Katz et al (Science Advances 2022; 8: 33) directly compared SomaScan with OLINK and found considerable differences in proteomic studies done with these two technologies, with evidence supporting more reliable protein target specificity for the OLINK platform. It is therefore likely that the discrepancies observed between our findings and those reported by Yang et al. represent technological differences between antibody- and aptamer-based platforms, with the former showing higher specificity (Katz et al., 2022). The differences between our study and Yang et al could also partly be cohort dependent. We now discuss these issues in more detail in the revised version of the paper. The discussion section includes this text (page 25):

"The overall replication rate between the studies was therefore quite low. This suggest that although some pQTLs can be robustly replicated across distinct proteomics methods, aptamer- and antibody-based technologies largely provide non-overlapping information. A recent large-scale study directly compared the aptamer-based SomaScan technology with antibody-based PEA and found considerable differences in results (Katz et al, 2022), which is in accordance with our findings. Importantly, Katz et al found evidence supporting more reliable protein target specificity and a higher number of phenotypic associations for the PEA assays, used in the current study. The specificity of aptamer-based multiplex methods has also been discussed before(Christiansson et al, 2014; Joshi & Mayr, 2018). Applied together,

these technologies may help to increase our understanding of the genetic regulation of the CSF proteome. Another possibility is that the differences between results in our study and Yang et al could partly also be cohort dependent."

4 - In general, the result section is scattered and lacking overall focus. Makes it difficult to determine the theme throughout and at times reads as a laundry list of analyses and statistical test. A reorganization of this section with a common theme

Answer: We have thoroughly reorganized the results section. We have moved up the sections on Mendelian Randomization and moved down – towards the end of the results – the sections on the GMNC/OSTN region and adjustment for ventricule volume. In our opinion, this creates a more logical flow and theme throughout the Results section. In the revised version of the paper, we have also focused on cis-pQTLs findings, and findings that were significant after Bonferroni correction for multiple comparisons (with less emphasis on trans-pQTL findings and findings that were not significant when correcting for multiple comparisons). We have also deleted a few lines which repeated information from the Methods or Discussion. In our opinion, these changes make the results section more focused.

Comment: Discussion:
1 - not enough attention is paid to the discrepancy with existing pQTL studies. Why so few overlaps? Is that overlap expected simply by chance?

Answer: We have expanded the discussion about the discrepancy. We have included a new section for a comparison with MR analyses in Yang et al. This text is included in the paper (page 19):

"We directly compared our MR findings with the findings in Yang et al (Yang et al, 2021) which included MR findings for CSF pQTLs and the outcomes AD, PD, ALS, stroke and FTD. Among our gene-level findings, the causal association between CD33 (also known as Siglec-3) and AD was replicated between the studies. The other MR findings were unique to our study and to those reported by Yang et al, respectively."

We have also added these text sections to the discussion (page 23):
"We note that another recent CSF pQTL study also implicated CD33 for AD by MR analysis(Yang et al, 2021), while several other proteins were uniquely implicated in this study versus in Yang et al.. "

See also the response above to comment Results#3.

2 - the significance of such a study is not made clear in the discussion. How does this lead to therapeutics? Are any new targets identified? Given that well known targets (e.g. APOE) are identified, does this add anything significant?

Answer: The well-known targets are encouraging as positive controls in this study, e.g. links between APOE and AD. The other proteins and genes identified here (most of which had not been identified in prior CSF pQTL studies) are potential candidates for novel treatments. We highlight novelty for drug development in Table 3. For example, the finding that GPNBM was causative for PD makes this a very attractive target for drug development. We note that this is also supported by a very recent paper in Science, which highlighted the role of GPNMB for alpha-synuclein pathology in PD (Diaz-Ortis et al, Science 2022). We also note that there

have been recent reports of increased levels of MMP-10 in Alzheimer's disease, and our MR findings suggest that this could be explored as a drug target. Literature searches on our other MR hits in ALS, epilepsy and depression/bipolar disease also suggested that the identified proteins could be relevant to pursue further as potential treatment targets, as explained in the text below.

We added this section to the paper (page 22-23):
"Several CSF proteins identified in the MR analysis for AD (GRN), preclinical AD (MMP-10), and PD (CD38 and GMBP) were also validated with observational differences between patients and controls. We did not find altered levels for CD33 or APOE (for AD) or ADAM15 (for PD). Hypothetically, this may be disease-stage dependent, since causal biomarkers may not always show cross-sectional differences during the later stages of the disease. We note that another recent CSF pQTL study also implicated CD33 for AD by MR analysis(Yang et al, 2021), while several other proteins were uniquely implicated in this study versus in Yang et al. The proteins identified in the MR analyses may be relevant to pursue as new treatment targets, and we note that candidate treatments are in development for some of these already. In Table 3 we list available drug candidates (proposed for non-neurological indications), which could potentially be explored for the neurological diseases identified in this study. Notably, several findings from our MR analysis are not listed already as drug targets and new be particularly interesting to explore as new targets. One particularly interesting candidate may be GPNMB in PD. GPNMB (Glycoprotein nonmetastatic melanoma protein B) is a transmembrane glycoprotein, which has been studied before in cancer and inflammation(Saade et al, 2021). Its potential role in PD pathogenesis and links to aggregation of α-synuclein has recently been highlighted (Diaz-Ortiz et al, 2022). Regarding the role of MMP-10 (matrix metalloproteinase 10) in preclinical AD, we note that previous studies found increased levels of CSF MMP-10 in AD, as well as increased rates of disease progression in those with high CSF MMP-10 levels at baseline (Martino Adami et al, 2022). Regarding the MR findings of CD33 (SIGLEC-3) in AD and SIGLEC-9 in ALS, we note that several members of the SIGLEC protein family have been implicated for roles in neurodegenerative diseases, especially due to neuroinflammatory properties(Siddiqui et al, 2019). The MR analyses also implicated FLRT2 and RARRES2 in epilepsy. To our knowledge, FLRT2 (fibronectin leucine-rich transmembrane protein 2) has not been described extensively in relation to epilepsy or seizures, but there is evidence that this cell adhesion molecule is involved in neuronal development, including development of inhibitory cortical circuits (Fleitas et al, 2021). RARRES2 (retinoic acid receptor responder protein 2, also known as chemerin) is described as having chemotactic properties during inflammatory responses and serum RARRES2 levels were associated with severity of seizures in children with idiopathic epilepsy(Elhady et al, 2018). CTSF (cathepsin F), implicated here for depression and bipolar disease, is a lysosomal protein which is involved in the pathogensis of some types of neuronal ceroid lipofuscinosis (Smith et al, 2013).

Another novelty is that no previous large-scale CSF pQTL study has utilized OLINK proteomics data. As we have discussed extensively above this is an important addition to previous published papers using aptamer-based methods. We also would like to highlight the unique integration of structural MRI in this study, where we show the importance of ventricular volume for CSF protein levels and their associations with genetic variants. This provides new explanations to recently described puzzling findings on links between genetic variants and CSF proteins (see above in our response to reviewer#2, where we describe our findings in the context of the recent publication by Jansen et al, 2022). We now conclude the paper with this section (page 27):

“The main novelties included the use of highly specific proximity extension assays in a large cohort, and the integration of brain volumetrics to account for confounding factors. New possible treatment targets for several neurological diseases were nominated.”

8th Nov 2022

Dear Dr. Mattsson-Carlgren,

Thank you for sending us your revised manuscript. We have now received the comments from the two referees who were asked to re-review your manuscript. As you will see below, the referees are satisfied with the modifications and think the study is now suitable for publication.

Before we can formally accept your manuscript, we would ask you to address the following editorial-level issues:

1. Please provide up to 5 keywords and incorporate them into the main text.

2. Remove the yellow color font.

3. The corresponding author should be defined on the manuscript title page.

4. Remove the author contribution section from the manuscript. We encourage you to use free text boxes in the online system instead.

5. Update the "Web Resources" heading to "For more information".

6. Please move the paper explained to the main manuscript file.

7. The current HTML graphs (Figure EV1,4,5) have to be converted to another image file format(.eps, .tif,. jpg). Please update the legends for the affected figures (including figure 2) accordingly.

The interactive plots should be uploaded as Source Data for these three figures. Please upload the interactive plots as a single ZIP file containing all the three Source Data files for figures EV1,4,5. Within the ZIP file, the Source Data should be included in individual folders pertaining to the figure that the Source Data is for.
More information regarding Source data formatting can be found here: More information can be found here:
https://www.embopress.org/page/journal/17574684/authorguide#sourcedata

8. We updated our journal's competing interests policy in January 2022 and request authors to consider both actual and perceived competing interests. Please review the policy https://www.embopress.org/competing-interests and update your competing interests if necessary.
Please use the heading "Disclosure statement and competing interests".

9. Funding information seems to be incomplete. Please note that funding sources and project numbers where available, should be in both manuscript and the online system.

10. Data availability: Please use the following statement: Anonymized data will be shared by request from a qualified academic investigator and as long as data transfer is in agreement with EU legislation on the general data protection regulation and decisions by the Ethical Review Board of Sweden and Region Skåne, which should be regulated in a material transfer agreement.

11. Please provide a striking image or visual abstract to illustrate your article as a PNG file 550 px wide x 300-600 px high.

12. The Material and Methods section should be placed after the Discussion.

13. Appendix
- Legends need updating to "Appendix Figure S1," etc.
- Methods: A4 genetic analyses should be merged with Methods in the main manuscript.

14. EV tables
- All the EV tables (except for EV2 and EV13) are rather large. Please update them to EV Datasets. EV Datasets need to be uploaded individually, with each legend inserted into each file in a separate sheet.
- Tables EV2 and EV13 should be renamed "Table EV1" and "Table EV2"
- Please update all corresponding callouts accordingly.

15. I have only slightly shortened the synopsis text as we have strict word limit(see attached). Please let me know if fine as is or if you would like to introduce further modifications.

Please submit your revised manuscript within two weeks. I look forward to seeing a revised form of your manuscript as soon as possible.

Sincerely,
Jingyi

Jingyi Hou
Editor
EMBO Molecular Medicine

***** Reviewer's comments *****

Referee #1 (Remarks for Author):

The authors have addressed my comments.

Referee #3 (Comments on Novelty/Model System for Author):

There is no model used here - just human data.

Referee #3 (Remarks for Author):

I believe that this revised manuscript is significantly improved from the original - and the responses to the initial review were done thoughtfully. I think that this has improved the technical quality of the work, particularly as it address the comparison with previous studies in this area. The acknoweldgements of some of the limitations as it relates to MR were a good addition. At this point it feels suitable for publication.

14th Nov 2022

Dear Dr. Mattsson-Carlgren,

I am pleased to inform you that your manuscript is accepted for publication and is now being sent to our publisher to be included in the next available issue of EMBO Molecular Medicine.

We would like to remind you that as part of the EMBO Publications transparent editorial process initiative, EMBO Molecular Medicine will publish a Review Process File online to accompany accepted manuscripts. If you do NOT want the file to be published or would like to exclude figures, please immediately inform the editorial office via e-mail.

Please read below for additional IMPORTANT information regarding your article, its publication and the production process.

Congratulations on your interesting work,

Jingyi

Jingyi Hou
Editor
EMBO Molecular Medicine

EMBO Press Author Checklist

Corresponding Author Name: Niklas Mattsson-Carlgren	
Journal Submitted to: EMBO Molecular Medicine	
Manuscript Number: EMM-2022-16359	

USEFUL LINKS FOR COMPLETING THIS FORM
The EMBO Journal - Author Guidelines
EMBO Reports - Author Guidelines
Molecular Systems Biology - Author Guidelines
EMBO Molecular Medicine - Author Guidelines

Reporting Checklist for Life Science Articles (updated January 2022)
This checklist is adapted from Materials Design Analysis Reporting (MDAR) Checklist for Authors. MDAR establishes a minimum set of requirements in transparent reporting in the life sciences (see Statement of Task: 10.31222/osf.io/9sm4x). Please follow the journal's guidelines in preparing your manuscript.
Please note that a copy of this checklist will be published alongside your article.

Abridged guidelines for figures
1. Data
The data shown in figures should satisfy the following conditions:
- the data were obtained and processed according to the field's best practice and are presented to reflect the results of the experiments in an accurate and unbiased manner.
- ideally, figure panels should include only measurements that are directly comparable to each other and obtained with the same assay.
- plots include clearly labeled error bars for independent experiments and sample sizes. Unless justified, error bars should not be shown for technical replicates.
- if n<5, the individual data points from each experiment should be plotted. Any statistical test employed should be justified.
- Source Data should be included to report the data underlying figures according to the guidelines set out in the authorship guidelines on Data Presentation.

2. Captions
Each figure caption should contain the following information, for each panel where they are relevant:
- a specification of the experimental system investigated (eg cell line, species name).
- the assay(s) and method(s) used to carry out the reported observations and measurements.
- an explicit mention of the biological and chemical entity(ies) that are being measured.
- an explicit mention of the biological and chemical entity(ies) that are altered/varied/perturbed in a controlled manner.
- the exact sample size (n) for each experimental group/condition, given as a number, not a range;
- a description of the sample collection allowing the reader to understand whether the samples represent technical or biological replicates (including how many animals, litters, cultures, etc.).
- a statement of how many times the experiment shown was independently replicated in the laboratory.
- definitions of statistical methods and measures:
  - common tests, such as t-test (please specify whether paired vs. unpaired), simple χ2 tests, Wilcoxon and Mann-Whitney tests, can be unambiguously identified by name only, but more complex techniques should be described in the methods section;
  - are tests one-sided or two-sided?
  - are there adjustments for multiple comparisons?
  - exact statistical test results, e.g., P values = x but not P values < x;
  - definition of 'center values' as median or average;
  - definition of error bars as s.d. or s.e.m.

Please complete ALL of the questions below.
Select "Not Applicable" only when the requested information is not relevant for your study.

Materials

Newly Created Materials	Information included in the manuscript?	In which section is the information available? (Reagents and Tools Table, Materials and Methods, Figures, Data Availability Section)
New materials and reagents need to be available; do any restrictions apply?	Not Applicable	

Antibodies	Information included in the manuscript?	In which section is the information available? (Reagents and Tools Table, Materials and Methods, Figures, Data Availability Section)
For **antibodies** provide the following information:<br>- Commercial antibodies: RRID (if possible) or supplier name, catalogue number and or/clone number<br>- Non-commercial: RRID or citation	Yes	Supplier name is provided for OLINK assays

DNA and RNA sequences	Information included in the manuscript?	In which section is the information available? (Reagents and Tools Table, Materials and Methods, Figures, Data Availability Section)
**Short novel DNA or RNA including primers, probes**: provide the sequences.	Not Applicable	

Cell materials	Information included in the manuscript?	In which section is the information available? (Reagents and Tools Table, Materials and Methods, Figures, Data Availability Section)
**Cell lines:** Provide species information, strain. Provide accession number in repository **OR** supplier name, catalog number, clone number, and/**OR** RRID.	Not Applicable	
**Primary cultures:** Provide species, strain, sex of origin, genetic modification status.	Not Applicable	
Report if the cell lines were recently **authenticated** (e.g., by STR profiling) and tested for mycoplasma contamination.	Not Applicable	

Experimental animals	Information included in the manuscript?	In which section is the information available? (Reagents and Tools Table, Materials and Methods, Figures, Data Availability Section)
**Laboratory animals or Model organisms:** Provide species, strain, sex, age, genetic modification status. Provide accession number in repository **OR** supplier name, catalog number, clone number, **OR** RRID.	Not Applicable	
**Animal observed in or captured from the field:** Provide species, sex, and age where possible.	Not Applicable	
Please detail **housing and husbandry conditions**.	Not Applicable	

Plants and microbes	Information included in the manuscript?	In which section is the information available? (Reagents and Tools Table, Materials and Methods, Figures, Data Availability Section)
**Plants:** provide species and strain, ecotype and cultivar where relevant, unique accession number if available, and source (including location for collected wild specimens).	Not Applicable	
**Microbes:** provide species and strain, unique accession number if available, and source.	Not Applicable	

Human research participants	Information included in the manuscript?	In which section is the information available? (Reagents and Tools Table, Materials and Methods, Figures, Data Availability Section)
If collected and within the bounds of privacy constraints report on age, sex and gender or ethnicity for all study participants.	Yes	

Core facilities	Information included in the manuscript?	In which section is the information available? (Reagents and Tools Table, Materials and Methods, Figures, Data Availability Section)
If your work benefited from core facilities, was their service mentioned in the acknowledgments section?	Not Applicable	

Design

Study protocol	Information included in the manuscript?	In which section is the information available? (Reagents and Tools Table, Materials and Methods, Figures, Data Availability Section)
If study protocol has been **pre-registered, provide DOI in the manuscript**. For clinical trials, provide the trial registration number **OR** cite DOI.	Not Applicable	
Report the **clinical trial registration number** (at ClinicalTrials.gov or equivalent), where applicable.	Yes	Materials and Methods

Laboratory protocol	Information included in the manuscript?	In which section is the information available? (Reagents and Tools Table, Materials and Methods, Figures, Data Availability Section)
Provide DOI OR other citation details if **external detailed step-by-step protocols** are available.	Not Applicable	

Experimental study design and statistics	Information included in the manuscript?	In which section is the information available? (Reagents and Tools Table, Materials and Methods, Figures, Data Availability Section)
Include a statement about **sample size** estimate even if no statistical methods were used.	Yes	Materials and Methods
Were any steps taken to minimize the effects of subjective bias when allocating animals/samples to treatment (e.g. **randomization procedure**)? If yes, have they been described?	Not Applicable	
Include a statement about **blinding** even if no blinding was done.	Yes	Materials and Methods
Describe **inclusion/exclusion criteria** if samples or animals were excluded from the analysis. Were the criteria pre-established?<br><br>If sample or data points were omitted from analysis, report if this was due to attrition or intentional exclusion and provide justification.	Yes	Materials and Methods
For every figure, are **statistical tests** justified as appropriate? Do the data meet the assumptions of the tests (e.g., normal distribution)? Describe any methods used to assess it. Is there an estimate of variation within each group of data? Is the variance similar between the groups that are being statistically compared?	Yes	Figures

Sample definition and in-laboratory replication	Information included in the manuscript?	In which section is the information available? (Reagents and Tools Table, Materials and Methods, Figures, Data Availability Section)
In the figure legends: state number of times the experiment was **replicated** in laboratory.	Not Applicable	
In the figure legends: define whether data describe **technical or biological replicates**.	Not Applicable	

Ethics

Ethics	Information included in the manuscript?	In which section is the information available? (Reagents and Tools Table, Materials and Methods, Figures, Data Availability Section)
Studies involving **human participants**: State details of **authority granting ethics approval** (IRB or equivalent committee(s), provide reference number for approval.	Yes	Material and Methods
Studies involving **human participants**: Include a statement confirming that **informed consent** was obtained from all subjects and that the experiments conformed to the principles set out in the WMA Declaration of Helsinki and the Department of Health and Human Services Belmont Report.	Yes	Material and Methods
Studies involving **human participants:** For publication of **patient photos**, include a statement confirming that consent to publish was obtained.	Not Applicable	
Studies involving experimental **animals**: State details of **authority granting ethics approva**l (IRB or equivalent committee(s), provide reference number for approval. Include a statement of compliance with ethical regulations.	Not Applicable	
Studies involving **specimen and field samples:** State if relevant **permits** obtained, provide details of authority approving study; if none were required, explain why.	Not Applicable	

Dual Use Research of Concern (DURC)	Information included in the manuscript?	In which section is the information available? (Reagents and Tools Table, Materials and Methods, Figures, Data Availability Section)
Could your study fall under dual use research restrictions? Please check biosecurity documents and list of **select agents and toxins** (CDC): https://www.selectagents.gov/sat/list.htm	Not Applicable	
If you used a select agent, is the security level of the lab appropriate and reported in the manuscript?	Not Applicable	
If a study is subject to dual use research of concern regulations, is the name of the **authority granting approval and reference number** for the regulatory approval provided in the manuscript?	Not Applicable	

Reporting

The MDAR framework recommends adoption of discipline-specific guidelines, established and endorsed through community initiatives. Journals have their own policy about requiring specific guidelines and recommendations to complement MDAR.

Adherence to community standards	Information included in the manuscript?	In which section is the information available? (Reagents and Tools Table, Materials and Methods, Figures, Data Availability Section)
State if relevant guidelines or checklists (e.g., **ICMJE, MIBBI, ARRIVE, PRISMA**) have been followed or provided.	Not Applicable	
For **tumor marker prognostic studies,** we recommend that you follow the **REMARK** reporting guidelines (see link list at top right). See author guidelines, under 'Reporting Guidelines'. Please confirm you have followed these guidelines.	Not Applicable	
For **phase II and III randomized controlled trials**, please refer to the **CONSORT** flow diagram (see link list at top right) and submit the CONSORT checklist (see link list at top right) with your submission. See author guidelines, under 'Reporting Guidelines'. Please confirm you have submitted this list.	Not Applicable	

Data Availability

Data availability	Information included in the manuscript?	In which section is the information available? (Reagents and Tools Table, Materials and Methods, Figures, Data Availability Section)
Have **primary datasets** been deposited according to the journal's guidelines (see 'Data Deposition' section) and the respective accession numbers provided in the Data Availability Section?	Yes	Materials and Methods
Were **human clinical and genomic datasets** deposited in a public access-controlled repository in accordance to ethical obligations to the patients and to the applicable consent agreement?	Not Applicable	
Are **computational models** that are central and integral to a study available without restrictions in a machine-readable form? Were the relevant accession numbers or links provided?	Not Applicable	
If publicly available data were reused, provide the respective **data citations in the reference list**.	Not Applicable	
